# Spatiotemporal evolution of AML immune microenvironment remodeling and RNF149-driven drug resistance through single-cell multidimensional analysis

**DOI:** 10.1186/s12967-023-04579-5

**Published:** 2023-10-27

**Authors:** Xin Wu, Zhongguang Wu, Woding Deng, Rong Xu, Chunmei Ban, Xiaoying Sun, Qiangqiang Zhao

**Affiliations:** 1 Department of spine surgery, Third Xiangya Hospital, Central South University, Changsha, 410013 Hunan China; 2 Department of Clinical Laboratory Medicine, The Eighth Affiliated Hospital, Sun Yat-sen University, Shenzhen, 518033 Guangdong P.R. China; 3 Xiangya School of Medicine, Central South University, Changsha, 410013 Hunan China; 4 Department of Pathology, The First People’s Hospital of Changde City, Changde, 415003 Hunan China; 5 Department of Hematology, The People’s Hospital of Liuzhou City, Guangxi, 545026 People’s Republic of China; 6 The First Hospital of Sun Yat-sen University, Guangzhou, 510080 Guangdong China; 7 School of Nursing, Sun Yat-sen University, Guangzhou, 528406 China; 8 Department of Hematology, The Qinghai Provincial People’s Hospital, Xining, 810007 People’s Republic of China

**Keywords:** Acute myeloid leukaemia, Single-cell sequencing, Bone marrow microenvironment, Immune cells, RNF149

## Abstract

**Background:**

The composition of the bone marrow immune microenvironment in patients with acute myeloid leukaemia (AML) was analysed by single-cell sequencing and the evolutionary role of different subpopulations of T cells in the development of AML and in driving drug resistance was explored in conjunction with E3 ubiquitin ligase-related genes.

**Methods:**

To elucidate the mechanisms underlying AML-NR and Ara-C resistance, we analyzed the bone marrow immune microenvironment of AML patients by integrating multiple single-cell RNA sequencing datasets. When compared to the AML disease remission (AML-CR) cohort, AML-NR displayed distinct cellular interactions and alterations in the ratios of CD4^+^T, Treg, and CD8^+^T cell populations.

**Results:**

Our findings indicate that the E3 ubiquitin ligase RNF149 accelerates AML progression, modifies the AML immune milieu, triggers CD8^+^T cell dysfunction, and influences the transformation of CD8^+^ Navie.T cells to CD8^+^T_Exh_, culminating in diminished AML responsiveness to chemotherapeutic agents. Experiments both in vivo and in vitro revealed RNF149’s role in enhancing AML drug-resistant cell line proliferation and in apoptotic inhibition, fostering resistance to Ara-C.

**Conclusion:**

In essence, the immune microenvironments of AML-CR and AML-NR diverge considerably, spotlighting RNF149’s tumorigenic function in AML and cementing its status as a potential prognostic indicator and innovative therapeutic avenue for countering AML resistance.

**Graphical Abstract:**

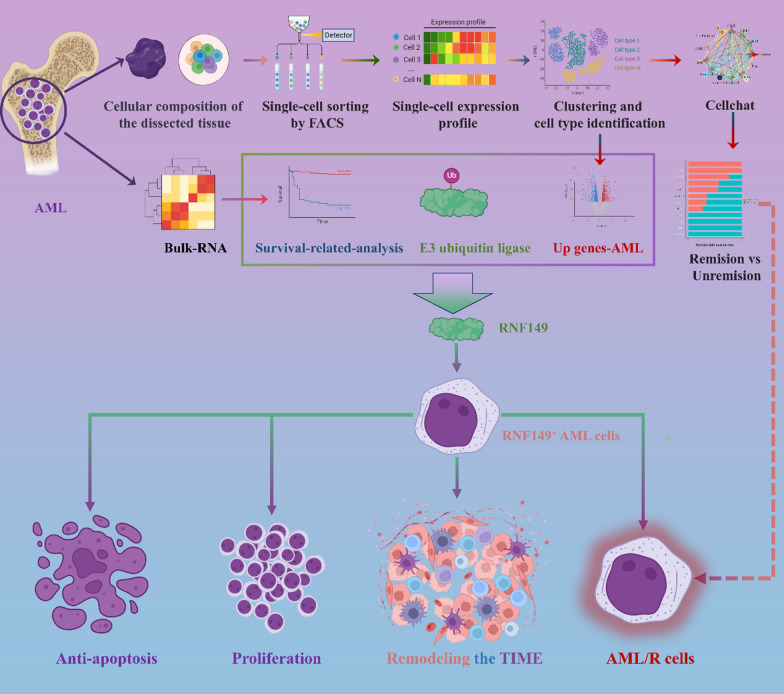

**Supplementary Information:**

The online version contains supplementary material available at 10.1186/s12967-023-04579-5.

## Introduction

Acute myeloid leukemia (AML) is a highly heterogeneous hematologic malignancy marked by the clonal proliferation of myeloid progenitor cells [1]. Globally, AML ranks among the top ten prevalent malignant tumors, with its incidence witnessing a consistent rise over recent decades [2]. Of all acute leukemia types, AML exhibits the lowest survival duration, registering a five-year survival rate below 30% [3]. Therapeutic interventions such as the “3^+^7” regimen, allogeneic hematopoietic stem cell transplantation, and molecular-targeted drugs addressing specific gene mutations and epigenetic anomalies have demonstrated potential in enhancing clinical prognosis and prolonging survival [4]. Nonetheless, due to AML’s intricate pathogenesis, these therapeutic advancements possess inherent limitations [5]. Consequently, the identification of novel molecular targets for AML, coupled with a deeper comprehension of the disease and the innovation of targeted therapeutic modalities, could significantly address these challenges, thereby amplifying patient recovery and longevity.

AML’s emergence and progression are substantially influenced by the bone marrow (BM) microenvironment [5]. Research indicates that both genetic and phenotypic alterations within this microenvironment bolster leukemia progression by fostering leukemia cell growth and opposing chemotherapy [6]. Recently, immunotherapy has marked notable achievements in treating lymphocytic leukemia and an array of solid tumors, positioning it as a cornerstone in cancer therapeutics [7–9]. Prevailing studies intimate that the immunological equilibrium within the bone marrow microenvironment is pivotal in AML’s immunotherapy and other treatments such as chemotherapy and targeted therapy [10]. Tumor cells manipulate and remodel the BM ecology, converting it into a configuration that shields the tumor, facilitating AML’s immune evasion, and bolstering resistance to treatments [11]. Within this aberrant BM setting, AML stem and progenitor cells retain their regenerative capacities, while residual lesions receive conducive sustenance, precipitating AML relapse [12]. Hence, a profound grasp of the bone marrow’s immune microenvironment is imperative to craft innovative therapeutic blueprints.

Chemoresistance to treatment in hematological malignancies presents a significant challenge in clinical care [13]. Recent studies have identified that intercellular interactions within the AML immisune microenvironment contribute to tumor resistance [14, 15]. Immune system homeostasis represents a multifaceted ecosystem, comprising diverse cell types, their secretory products (e.g., cytokines and chemokines), and other elements, characterized by marked heterogeneity, dynamism, and intricate intercellular relationships [16]. Both effector and inhibitory immune cells, along with stromal components, play roles at various stages in tumor cell proliferation, augmented drug resistance, and diminished anti-tumor immune responses [17]. As such, elucidating the mechanisms underlying the modulation of immune responses within the AML immune landscape is pivotal for counteracting immune resistance [18]. While specific immune cell subgroups, rather than individual cell types, are intricately linked to AML’s onset and progression [19]. The precise composition and nature of the AML immune microenvironment remain to be defined. Single-cell sequencing serves as a prominent tool for uncovering the heterogeneity and diversity of tumor cells by assessing individual cell transcription profiles [20]. This technique offers deeper insights into the composition and functionality of diverse immune cell subsets within the AML bone marrow niche, fostering a more profound comprehension of its immune microenvironment.

In this study, we discern the variations in the cellular makeup of the bone marrow immune microenvironment in AML patients using single-cell transcriptomic datasets (GSE116256, GSE130756, and GSE198681). Our findings shed light on the adaptive nature of immune cells and the inherent tumor heterogeneity in AML. Furthermore, our exploration of AML resistance, based on distinct cell phenotypes, augments the existing body of knowledge on the AML immune milieu and its correlation with chemoresistance.

## Materials and methods

### Data sources

AML bone marrow samples’ single-cell sequencing datasets were sourced from the Gene Expression Omnibus (GEO) database. Based on patient-specific clinical data, we selected the scRNA-seq datasets (GSE116256, GSE130756, and GSE198681). Clinical bulk RNA-seq samples with AML gene expression profiles and related survival data were also extracted from the GEO database, with GSE106291 and GSE71014 meeting the inclusion criteria.

### scRNA-seq data quality control and analysis

The Seurat R package (v4.1.3) facilitated the reading, quality assessment, dimensionality reduction, and clustering of scRNA-seq data. Cells with gene counts below 1000 or exceeding 9000, and those with a mitochondrial gene expression ratio surpassing 10%, were deemed low-quality and excluded. Subsequent normalization employed the “log-normalize” method. High variability genes for the forthcoming Principal Component Analysis (PCA) were determined using the FindVariableFeatures function. Initially, the top 10 principal components were identified for dimensionality reduction. Cell type clustering utilized the Seurat FindClusters function (resolution = 0.20), succeeded by UMAP and tSNE methods for further dimension reduction and visualization. The FindAllMarkers function identified cluster-specific genes. Clustree displayed inter-cluster relationships across various resolutions. Drawing from cell annotations, ClusterGVis showcased differential gene heatmaps and enrichment analysis. Visualization of marker genes incorporated tools like the Nebulosa Rpackage, scCustomize Rpackage, and the Ridgeplot function.

### Intercellular communication analysis

We employed CellChat (version 1.5.0) to analyze intercellular communication. Initially, we formed a CellChat object through the “createCellChat” function using the RNA expression matrix and cell data. We then performed downstream analyses utilizing the integrated expression of ligand-receptor (L-R) interactions, encompassing “Secreted Signaling,“ “ECM-Receptor,“ and “Cell-Cell Contact.“ The “computeCommunProb” function facilitated the calculation of communication probabilities and the inference of cellular interaction networks. For discerning global communication patterns, the “selectK” function was used with nPatterns set to 3 for both incoming and outgoing communication.

### Analysis of cell trajectory

Using the R packages Monocle2 and Monocle3, we conducted a pseudotemporal analysis to trace the branching developmental trajectories of CD8^+^ T cell subsets. We built single-cell developmental trajectories in a pseudotime sequence, grounded on genes differentially expressed between clusters. The double data rate tree (DDRTree) method was utilized for dimensionality reduction. Visualization was achieved with the plot_cell_trajectory function in Monocle, aligning cells along differentiation trajectories.

### E3 ubiquitin ligase-related gene survival analysis

We consolidated 2407 ubiquitination genes, derived from the annotations of the Ubiquitin and Ubiquitin-like Conjugation Database (IUUCD) (http://iuucd.biocuckoo.org/). We conducted a survival analysis for individual genes using the R package ‘survival’. Patients were categorized into high-expression and low-expression cohorts based on each gene’s cutoff values. The Log-Rank test determined the statistical significance of survival curves, considering curves with P < 0.05 as distinct.

### Pathway analysis

The FindMarkers function, utilizing the Wilcoxon algorithm, facilitates the comparison of cells from diverse samples within the same cell type to discern differentially expressed genes (DEGs). The criteria for screening include: |avg_log2FC| > 1 and P < 0.05. For varied cell types’ DEGs, clusterProfiler assists in conducting enrichment analysis via the Kyoto Encyclopedia of Genes and Genomes (KEGG). KEGG pathways with a significance level of P < 0.05 are chosen to elucidate the principal biological roles of the DEGs. The GSEA and GSEV analyses employ the R packages of GSEA and GSVA, respectively.

### Clinical cohort’s Bulk-RNA seq correlation

CibersortX and the mean expression of marker genes serve to deduce the infiltration scores of individual cell subtypes in the TARGET clinical cohort. The association between RNF149 and cell clusters is discerned through Spearman correlation analysis.

### Drug sensitivity (IC50) and RNF149 gene expression interrelation

The OncoPredict R package predicts drug responses in acute myeloid leukemia patients. It correlates the gene expression profile of tissues with the IC50 values from GDSC (https://www.cancerrxgene.org/) and the gene expression data from the Cancer Cell Line Encyclopedia (CCLE; https://portals.broadinstitute.org/ccle_legacy/home). In total, 198 drugs were assessed, and the association between drug IC50 and the RNF149 gene was evaluated using Spearman’s correlation. Significant correlations have a coefficient > 0.2 and an FDR < 0.05.

### Crafting drug-resistant cell strains

Following the methods in study [21], MOLM13 and MV4-11 cells are cultivated in IMDM medium supplemented with 10% fetal bovine serum, maintained at 37 °C in a 5% CO_2_ environment, and sub-cultured every 2–3 days. For drug-resistance establishment, the induction begins at IC50 of cytarabine. Once stable cellular proliferation is observed, the concentration is doubled. This process continues until cells sustain growth at cytarabine concentrations over 100 µmol/L^22^, indicative of cytarabine resistance.

### Fluorescent PCR quantification of mRNA levels

Cells are combined with 1 ml TRIzol and 200 µl of chloroform to extract total RNA. Following RNA isolation, a reverse transcription mix, as specified by Takara, Japan, is used, typically involving a 37 °C, 15-minute reaction and an 85 °C, 5-second reaction. The resultant cDNA functions as the PCR template, with primer specifics outlined in Additional file 1: Table S1. All samples and chemicals are maintained in a cold, dark environment. cDNA is diluted 3–5 fold before PCR, which involves 40 cycles of: 95 °C for 30 s; 60 °C for 5 s, followed by 60 °C for 34 s, concluding with a melting curve phase. Each target gene’s relative expression is determined using the 2^−ΔΔCt^ method with triplicate samples.

### siRNA transfection

Logarithmically growing cells are placed in 6-well plates. Upon reaching 70–80% confluency, transfection follows the Lip3000 protocol. Post a 6-h transfection, cells are nurtured in fresh medium for another 24 h. They are subsequently harvested for ensuing procedures. (Refer to Additional file 1: Table S1 for the si-RNF149 sequence).

### CCK-8 assay for evaluating the effects of RNF149 knockdown on cell viability

MOLM13/R and MV4-11/R cells were plated in a 96-well format at a concentration of 1 × 10^4^/ml, with each well containing a 100 µl cell suspension. Both cell lines were transfected with si-NC and si-RNF149, with triplicate wells for each condition. Following 24, 48, and 72-hour incubation periods, 10 µl of CCK-8 solution in complete medium was introduced and incubated for an additional 4 h. Absorbance was measured at OD450 using an ELISA reader. Data analysis was conducted based on dose-response curves with the aid of GraphPad Prism 7 software.

### Detection of apoptosis via flow cytometry

MOLM13/R and MV4-11/R cells, previously transfected with si-NC and si-RNF149, were washed in PBS and subsequently treated with 1× Binding Buffer. After centrifugation, the supernatant was removed. The cell pellet was resuspended in 100 µL of 1× Binding Buffer, treated with 10µL of Annexin V-FITC, and then incubated for 15 min in the dark at room temperature. Following a buffer wash and centrifugation, cells were treated with 5 µL PI and then analyzed for apoptosis using flow cytometry.

### Soft agar colony formation assay to evaluate cell proliferation

For the assay, a base layer containing 0.6% agarose was prepared and dispensed into a 6-well plate (0.5ml/well). Upon solidification, it was incubated at 37 ℃ with 5% CO_2_. The top layer, made of 0.4% agarose, contained logarithmically proliferating MOLM13/R and MV4-11/R cells, previously transfected with si-NC or si-RNF149. Post-centrifugation at 1000 rpm for 5 min, cells were resuspended in IMDM medium, counted, and appropriately diluted to achieve a concentration of 1000 cells/0.5 ml. Continuous incubation ensued at 37 ℃ in 5% CO_2_ for 10–14 days. Visible colonies were monitored, and upon their detection, incubation ceased. Colony count was undertaken using an inverted microscope, allowing for the calculation of the colony formation rate. The entire procedure was executed thrice.

### In vivo xenograft mouse experiment

6-week old female Balb/c-nude mice (6 in total) were randomly divided into 2 groups: knockdown group and control group, with 3 mice per group. MOLM13/R cells (1 × 10^7^ cells, 0.1 mL PBS) transfected with stable RNF149 knockdown (sh-RNF149 sequence see Additional file 1: Table S1) and control (sh-NC) were injected subcutaneously into the NSG mice. Mice from each group were intraperitoneally injected with cytarabine (at a dose of 250 mg/kg) continuously for 7 days. Tumor width and length were recorded every 3 days. Tumor volume was calculated as follows: Volume = (length × width^2^)/2. After 21 days, the mice were euthanized and the tumors weighed.

### Hematoxylin and eosin staining of tumor tissue

Paraffin sections of tumor tissue were successively placed in xylene and graded alcohols for deparaffinization to water. The tissues were then stained with hematoxylin for nuclei and eosin for cytoplasm. The slides were then dehydrated in graded alcohols and xylene and mounted with neutral gum. Observations and photographs were taken under a fluorescence microscope.

### TUNEL assay

Using an in-situ cell death detection kit with fluorescent markers, the apoptotic state of subcutaneous AML tumor tissues was determined via TUNEL staining. Frozen sections of mouse tumor tissues were prepared. After reaching room temperature, slides were washed twice with PBS. 50 mL of TdT and dUTP mixture was added to the slides and incubated in a humidified dark box at 37 °C for 60 min. Slides were then washed with PBS and mounted with an anti-fading solution. Detection was performed under a fluorescence microscope.

### Immunofluorescence staining

Paraffin-embedded slides were baked at 70 ℃ for 1 h, and then successively placed in xylene, absolute ethanol, 90% ethanol, 80% ethanol, 70% ethanol, and PBS for deparaffinization and rehydration. Antigen retrieval was performed in a pressure cooker with citrate antigen retrieval solution (pH = 6.0). After cooling, the retrieval solution was washed off with PBS. Sections were blocked at room temperature for 20 min, then incubated overnight at 4℃ with RNF149 antibody (diluted 1:1000 in antibody dilution buffer). After washing the slides 3 times with 0.1% PBST, they were incubated with Alexa Fluor 555 labeled IgG antibody (diluted 1:600 in antibody dilution buffer) at room temperature for 20 min. After washing off the antibodies with 0.1% PBST, slides were mounted with a mounting medium containing DAPI and observed under a fluorescence microscope.

### Statistical analysis using SPSS 23.0 software

Comparison between groups for normally distributed measurement data was done using the independent sample t-test. Non-normally distributed measurement data was compared using the Wilcoxon rank-sum test. A P-value less than 0.05 was considered statistically significant.

## Results

### Establishment of single-cell overview of AML bone marrow microenvironment

**Fig. 1:**
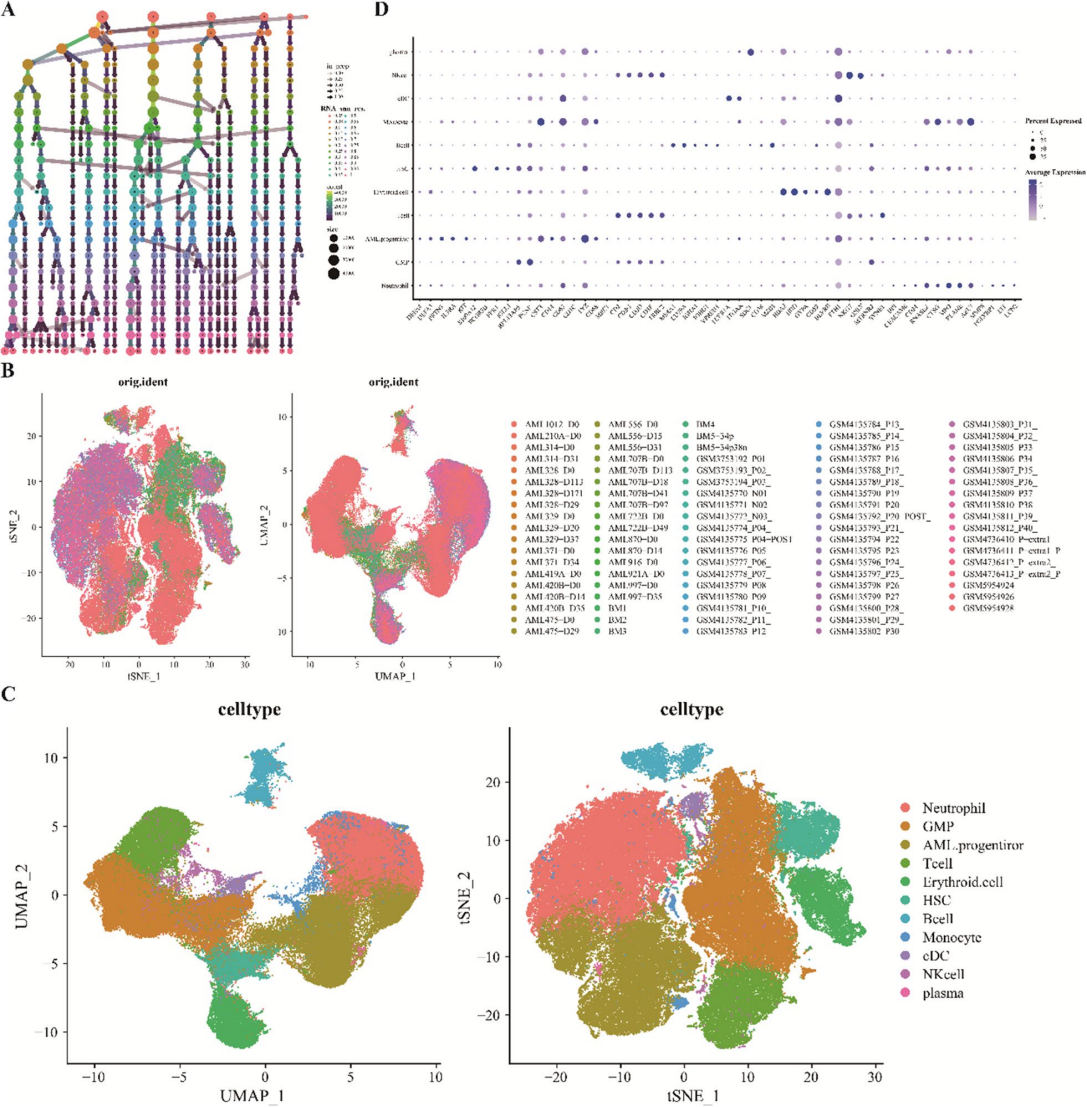
Transcriptomic profiling of individual AML cells. **A** Cell clustering across varying resolutions. **B** t-SNE and UMAP visualizations of 84 acute myeloid leukemia samples compared to 9 healthy control samples. **C** t-SNE and UMAP representations of 11 primary cell types identified within AML. **D** Dot plots highlighting the characteristic genes with high expression across 101 cell clusters. Dot size corresponds to the percentage of cells expressing specific markers, while the color gradient reflects the average expression level of these markers

Utilizing single-cell transcriptomic data from prior 10× Genomics sequencing (GSE116256, GSE130756, and GSE198681), we investigated cellular types and molecular signatures within AML. Initially, we excluded cells exhibiting elevated mitochondrial gene content. Subsequent dimensionality reduction was performed via analysis of highly variable features (HVGs) and principal component analysis (PCA) (Additional file 1: Fig. S1A–B). The effect of cell cycle genes on cellular clustering is noted (Additional file 1: Fig. S1C). We further assessed the relationship among mitochondrial-associated genes, nfeature, and ncount (Additional file 1: Fig. S1D–E). Detailed analyses regarding nfeature, ncount, and cell cycle-associated scores for every patient were provided (Additional file 1:  Fig. S1F–I). Following quality control measures and batch effect mitigation, a total of 121,383 cells were identified. Using clustree, the impact of varying clustering resolutions on cellular grouping was illustrated as a dendrogram (Fig. 1A). Upon inspecting t-SNE and UMAP visuals post batch-effect correction, a uniform cell distribution was evident, underscoring effective bias elimination (Fig. 1B). Leveraging established marker genes, we discerned 11 primary cell clusters: Neutrophil, HSCs, AML progenitors, GMPs, monocytes, T cells, B cells, plasma cells, conventional dendritic cells, and erythroid cells (Fig. 1C). High-expression gene profiles facilitated cell type identification within each cluster (Fig. 1D).

### Heterogeneity in acute myeloid leukemia cells

**Fig. 2:**
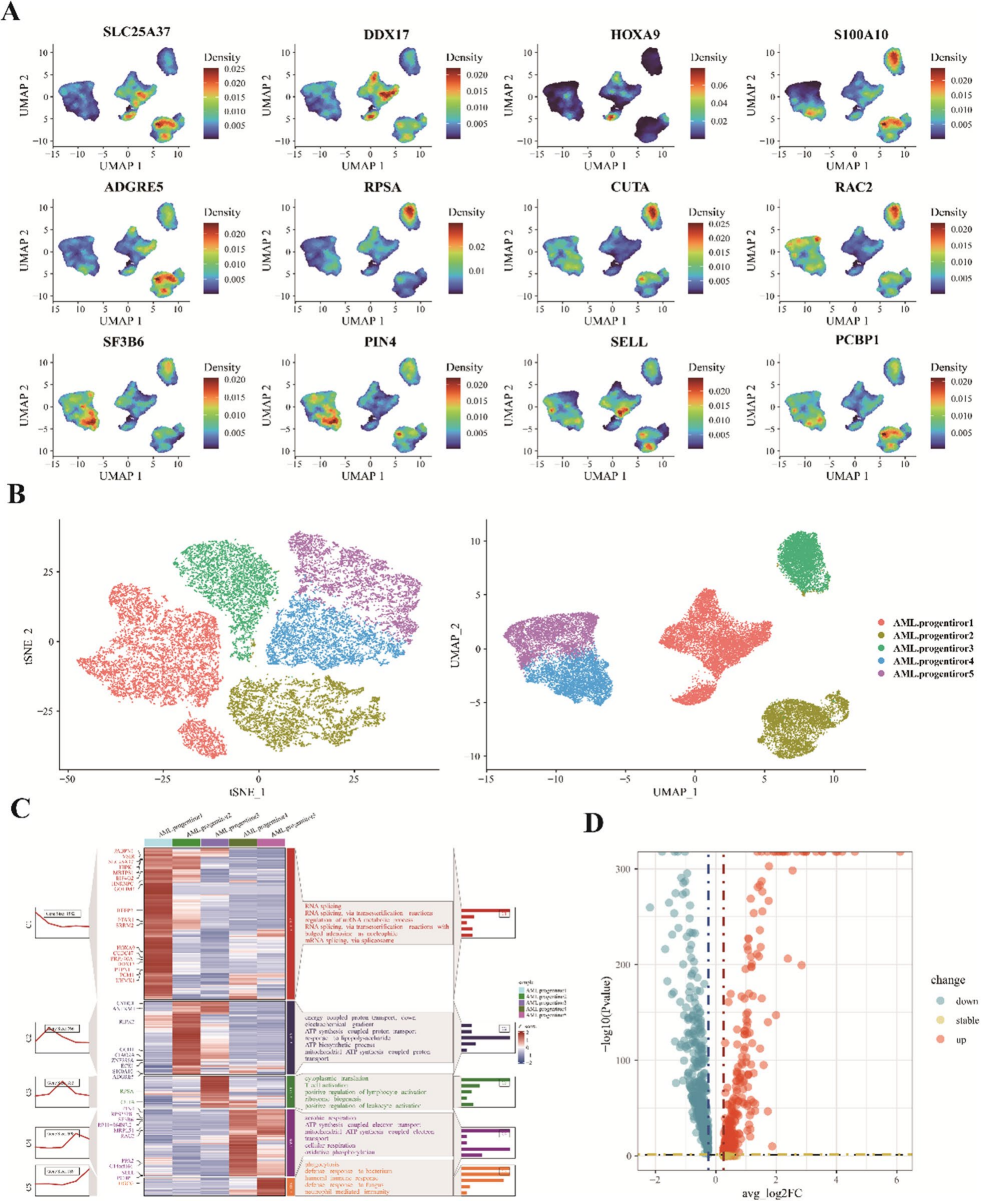
Heterogeneity in acute myeloid leukemia (AML) cells. **A** Marker gene profiles across distinct AML progenitor cell types; **B** Five main AML progenitor cell subgroups identified via UMAP analysis; **C** Expression heatmap and GO analysis for DEGs in the five AML progenitor subclusters; **D** Scatter plot comparing DEGs in post-chemotherapy remission AML cells to those not achieving remission

Through UMAP analysis and marker gene identification of acute myeloid leukemia (AML) cells (Fig. 2A), we discerned five distinct subgroups, designated as AML.progenitors1-5 (Fig. 2B). Each subgroup displayed unique transcriptomic profiles and pathway enrichments. Specifically, AML.progenitors1 primarily functions in RNA splicing, generating mature mRNAs capable of protein encoding—a pivotal mechanism for gene expression diversity. AML.progenitors2 is involved in cellular energy metabolism and immune response, emphasizing ATP synthesis and proton transport’s roles in energy regulation. AML.progenitors3 focuses on cellular protein synthesis, T-cell and lymphocyte activation, and ribosomal biosynthesis, all essential for regular biological functions and immune responses. AML.progenitors4 plays a vital role in cellular energy acquisition, particularly via mitochondrial oxidative phosphorylation, facilitating efficient ATP production. AML.progenitors5 is associated with immune defense mechanisms, emphasizing phagocytosis, bacterial and fungal defense, and neutrophil immune responses (Fig. 2C). In comparison to AML cells that entered remission post-chemotherapy, we noted a different expression pattern in those remaining in a non-remissive state (Fig. 2D). Our findings underscore the marked differences in AML cell clusters, highlighting the disease’s inherent heterogeneity.

### Subgroups of T lymphocytes

**Fig. 3:**
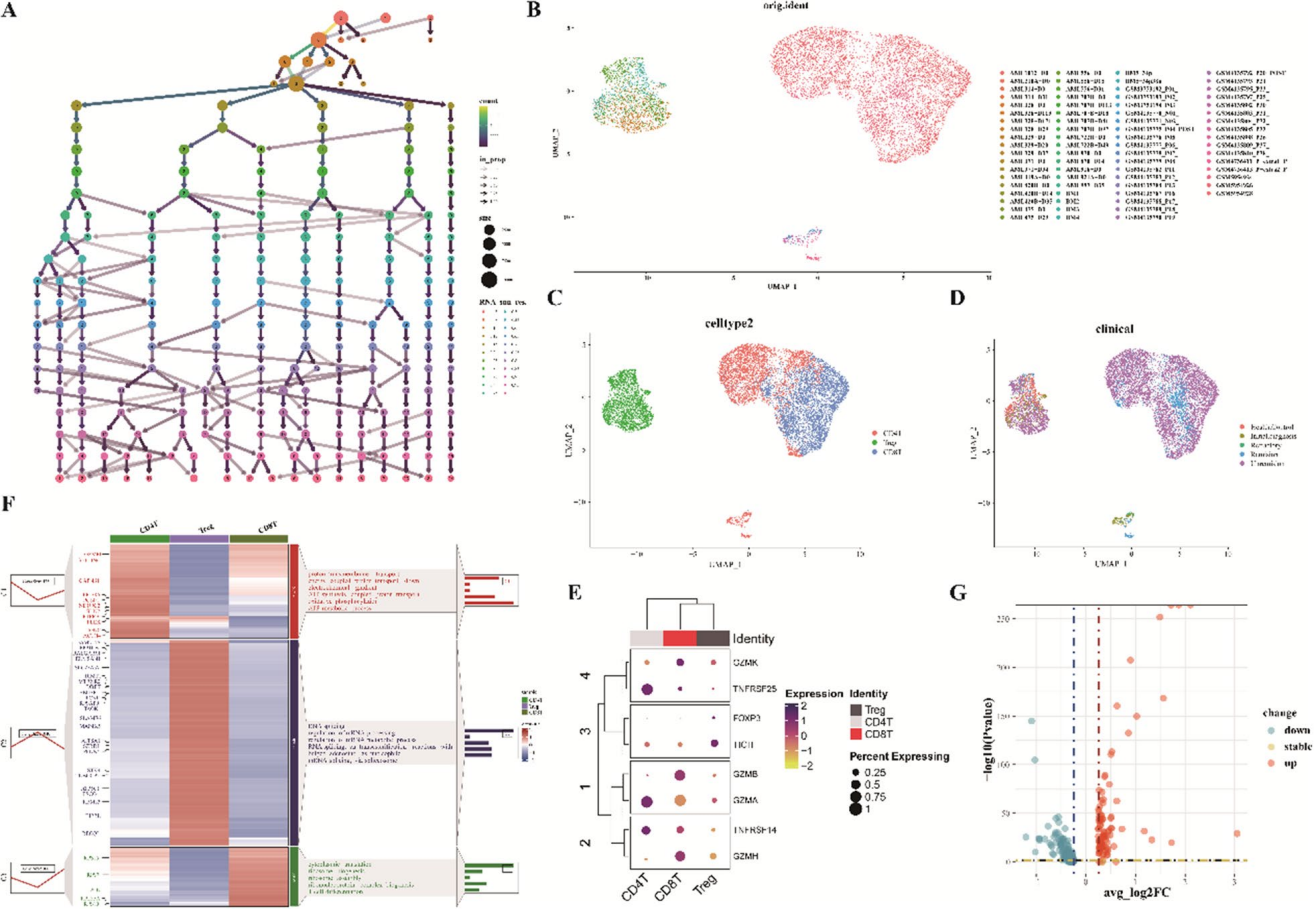
Comprehensive profile of T cells within the AML immune microenvironment. **A** Cell clustering across various resolutions; **B**–**D** UMAP visualizations for diverse samples, cell types, and clinical classifications; **E** Dot plot representation of canonical T cell-associated markers; **F** Heatmap of DEG expression and accompanying GO analysis for the three T cell subclusters; **G** Volcano plot contrasting differential gene expression in T cells post-chemotherapy remission with those not achieving remission (genes highlighted in red signify upregulation during relapse)

Immune cells within the bone marrow microenvironment critically influence the onset, progression, and clearance of AML. A disturbance in T cell immune homeostasis bears a notable association with AML. Analyzing alterations in T cell subgroups during various disease stages of AML is instrumental in predicting therapeutic outcomes and offers a foundational basis for identifying new immunotherapy targets. We determined the optimal resolution for subgrouping by evaluating its effects under varying resolutions (Fig. 3A). Examination of T lymphocytes via the UMAP plot revealed that the two-dimensional cell distribution does not demonstrate a discernible correlation among samples, signifying effective removal of batch effects (Fig. 3B). Three distinct subgroups were identified: CD4^+^T, Treg, and CD8^+^T cells (Fig. 3C). These cells predominantly appear in patients who didn’t achieve remission post-chemotherapy, though they are present in reduced quantities in those who did, as well as in refractory, newly diagnosed patients, and healthy controls (Fig. 3D). Dot plots of previously researched marker genes indicate their efficacy in cell subgrouping (Fig. 3E). Different T lymphocyte types exhibit distinct gene expressions and biological functions. The role of CD4^+^T cells predominantly involves sustaining intra-cellular metabolism and energy, bolstering their activation during immune responses, cytokine secretion, and the realization of various immune functions. Such processes are imperative for CD4^+^T cells’ optimal functioning within the immune system. Treg cells, in their association with RNA splicing and mRNA processing, possibly exert immunomodulatory effects through the regulation of specific genes. This may encompass adjustments in mRNA splicing, processing, and metabolism, subsequently influencing gene expression and immune cell functionality. CD8^+^T cells primarily specialize in protein synthesis and immunity. Processes like cytoplasmic translation, ribosomal biosynthesis, assembly, and ribonucleoprotein complex formation are essential for their immunological roles, ensuring adequate protein synthesis for their defense against pathogens and aberrant cells (Fig. 3F). In alignment with AML progenitor cells, T cells from post-chemotherapy remission and non-remission show pronounced transcriptomic disparities (Fig. 3G).

### Cell communication and global communication patterns in the acute myeloid leukemia tumor immune microenvironment

**Fig. 4:**
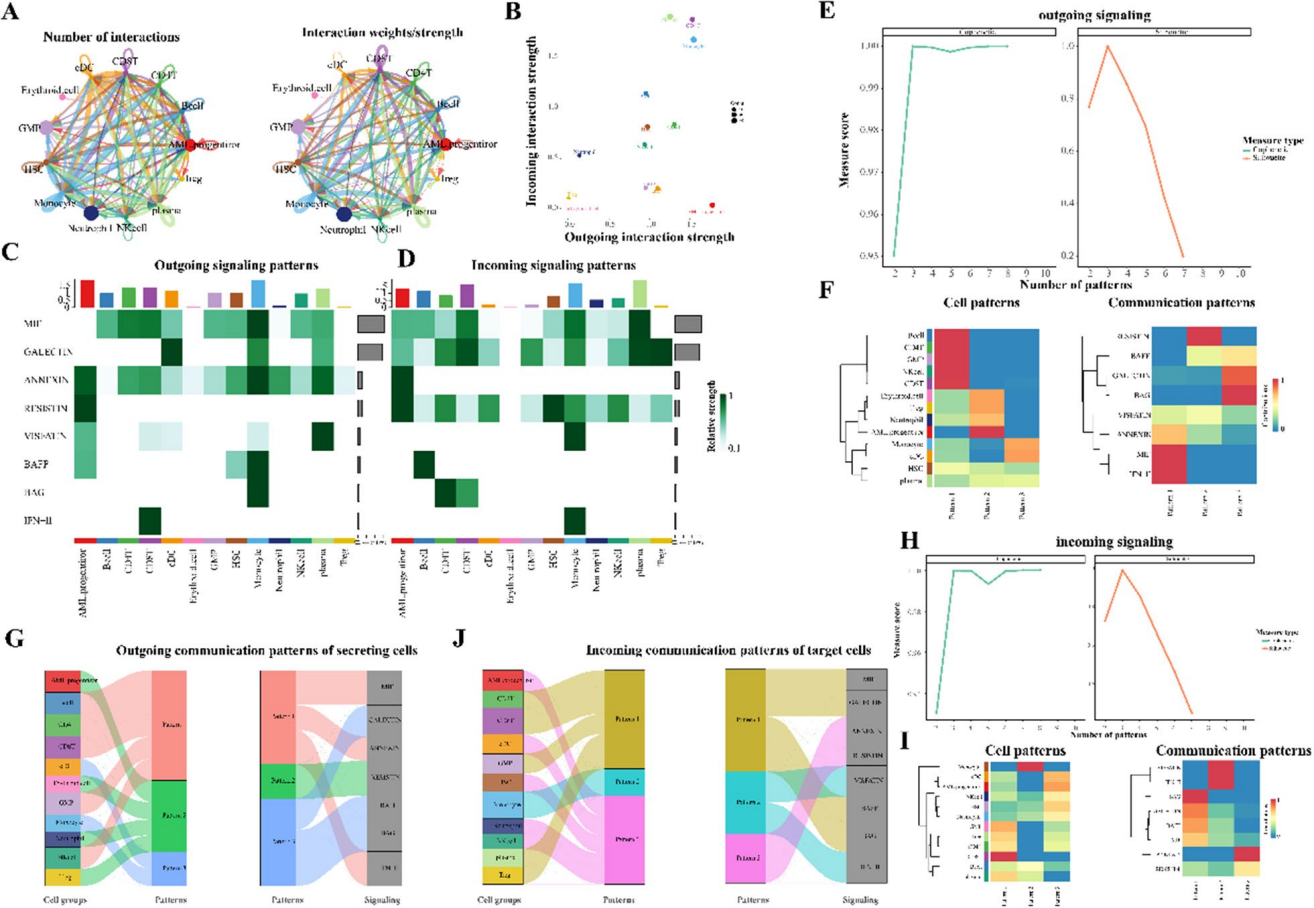
Detailed analysis of cell subpopulation communication and overarching cellular communication patterns. **A** Communication frequency and intensity among cell subpopulations; **B** 2D representation of primary emitters and receivers; **C**-**D** Key outgoing and incoming signal patterns; **E** Estimation of output pattern count using Cophenetic and Silhouette metrics; **F** Cell subpopulations and associated signaling pathways for output patterns; **G** River plot of secretion cell outgoing signal trends; **H** Estimation of input pattern count using Cophenetic and Silhouette metrics; **I** Cell subpopulations and associated signaling pathways for input patterns; **J** River plot depicting secretion cell incoming signal trends

The Tumor Microenvironment (TME) significantly influences various stages of tumor initiation, progression, metastasis, and treatment. Specifically, the immune microenvironment, shaped by immune cell infiltration, critically impacts the treatment outcomes and prognosis of AML. Our observations in AML indicate that cell subgroups demonstrate a high level of communication in terms of both quantity and intensity (Fig. 4A). Using a 2D representation, we identified dominant cell senders and receivers. The primary senders encompass AML progenitor cells, GMP cells, and cDC cells, whereas Plasma cells, CD8^+^T cells, and monocytes predominantly serve as receivers (Fig. 4B). Our research also unveiled the ability of AML to modulate other cell subgroups through diverse signaling pathways (Additional file 1: Fig. S2). In our examination of signal distribution among cell subgroups, MIF and GALECTIN emerged as the primary outgoing and incoming signals, respectively (Fig. 4C, D). As we delved deeper into the communication dynamics of each cell subgroup and pathway, a pressing question arose: how best to harmonize the functions of multiple cell assemblies and signaling pathways. Utilizing CellChat, we discerned global communication patterns by leveraging pattern recognition techniques. Employing Silhouette, we ascertained the number of output and input patterns (Fig. 4E, H), subsequently classifying cell subgroups and communication patterns into distinct categories (outputs: n = 4, inputs: n = 3) (Fig. 4F, I). We further illustrated the potential output patterns’ relationships with cell clusters and signaling pathways using a river plot (Fig. 4G, J). Our findings underscore the pivotal role of AML cells, CD4^+^T, Treg, and CD8^+^T cells in these global communication paradigms.

### Cell communication variations in acute myeloid leukemia patients resistant to chemotherapy

**Fig. 5:**
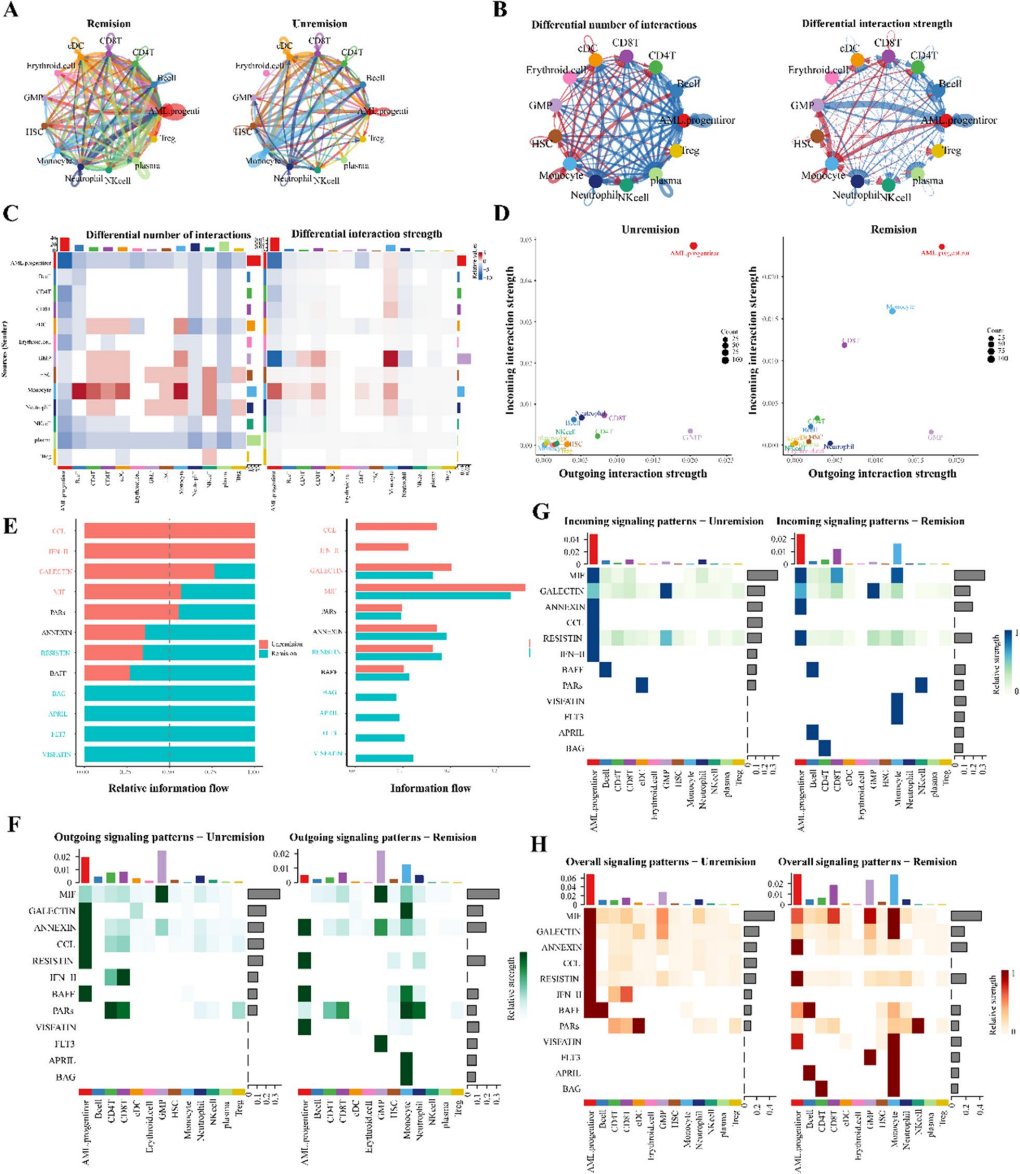
Cellular communication differences between AML patients post-chemotherapy remission and non-remission. **A** Cellular interaction counts during remission and non-remission; **B** Interaction variance in non-remission (red lines indicate high expression; blue indicate low expression); **C** Heatmap illustrating interaction variance in count and intensity; **D** Comparison of primary interaction sources and targets in a 2D space; **E** Overview of information flow in distinct signaling pathways; **F** Outgoing signal patterns for individual cell groups; **G** Incoming signal patterns for individual cell groups; **H** Overall signal patterns linked to each cell group

Chemotherapy stands as the predominant treatment for AML patients. Nonetheless, those resistant to this treatment approach face an escalated risk of disease progression, jeopardizing their overall well-being. Prior extensive studies indicate the role of cell communication in influencing disease phenotypes. Investigating the cell communication discrepancies between post-chemotherapy remission and non-remission patients is imperative to understand the susceptibility of Acute Myeloid Leukemia to chemotherapy. Figure 5 A illustrates that both cohorts, remission and non-remission, display profuse cell communication. The cellular communication networks in these groups differ markedly in interaction frequency and intensity (Fig. 5B). Utilizing heatmaps and 2D representations, we elucidate disparities in cell subgroup communications. Notably, AML progenitor cells, GMP cells, and CD8^+^T cells emerge as prominent senders and receivers across both groups (Fig. 5C, D). Delving deeper into the mechanisms of non-remission post-chemotherapy, our differential analysis revealed that specific pathways, including CCL, IFN-II, GALECTIN, and MIF, witness heightened expression in non-remission acute myeloid leukemia after chemotherapy (Fig. 5E). Moreover, a comparative analysis of these pathways and the modulation of ligand-receptor pairs suggests distinct communication patterns for AML patients in both categories (Fig. 5F–H, Additional file 1: Fig. S3).

### The regulatory significance of E3 ubiquitin ligase RNF149 in AML

**Fig. 6:**
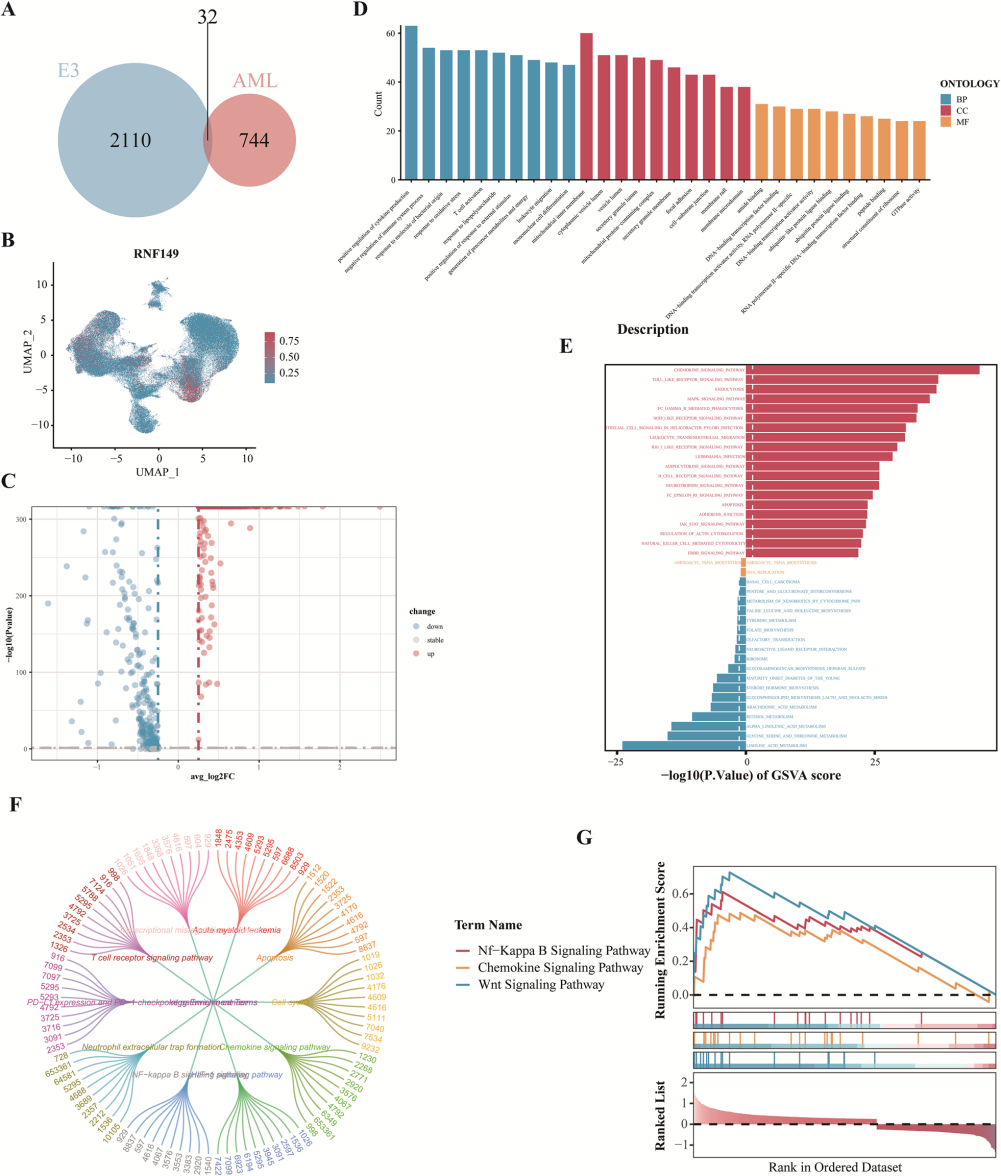
Regulatory significance of RNF149 in acute myeloid leukemia (AML). **A** Venn diagram illustrating the overlap between ubiquitin ligase E3 and genes highly expressed in AML progenitors; **B** UMAP representation of RNF149 expression; **C** Volcano plot highlighting genes differentially expressed at varying RNF149 levels; **D** Gene Ontology (GO) analysis for these genes; **E** Gene Set Variation Analysis (GSVA) for differentially expressed genes; **F** KEGG pathway analysis for these genes; **G** Gene Set Enrichment Analysis (GSEA) for differentially expressed genes

Proteins, as primary executors of biological activities, can undergo covalent modifications to interact with various molecules. These modifications can alter protein conformation, enzymatic activity, cellular localization, interactions, and stability, thereby playing crucial regulatory roles. Ubiquitination, a critical covalent protein modification, significantly influences all facets of standard cellular physiology. E3 ubiquitin ligases are pivotal enzymes in protein ubiquitination, recognizing specific substrates for ubiquitination. Exploring the link between E3 ubiquitin ligase and AML can offer insights for potential therapeutic targets. A comparison between 2110 E3 ubiquitin ligases from IUUCD and 744 differentially expressed genes in AML progenitor cells yielded 32 AML-E3s genes (Fig. 6A). Subsequent verification in the GEO AML database revealed RNF149’s strong prognostic prediction potential (Additional file 1:  Figs. S4 and S5). To ascertain RNF149’s influence on AML cells, AML progenitors were categorized based on RNF149 expression levels (Fig. 6B), and differential gene analysis results were represented using a volcano plot (Fig. 6C). GO analysis indicated a primary enrichment in tumor microenvironment and immune regulatory functions, hinting at RNF149’s role in immune system activation and regulation. Cellular components were predominantly associated with cellular structures and interactions, emphasizing RNF149’s significance in cellular signaling and transport (Fig. 6D). GSVA analysis revealed upregulated metabolic and functional pathways in cells with high RNF149 expression, touching on vital areas of cellular biology (Fig. 6E). KEGG pathway analysis was linked to cancer development and immune disorders, highlighting its relevance in cancer and immune disease treatment (Fig. 6F). GSEA analysis suggested that RNF149 high-expression cells might activate certain signaling pathways, potentially influencing tumor progression and immune responses (Fig. 6G). Collectively, these findings underscore RNF149’s role in modulating the immune microenvironment in AML.

### RNF149’s role in modifying the tumor microenvironment of AML

**Fig. 7:**
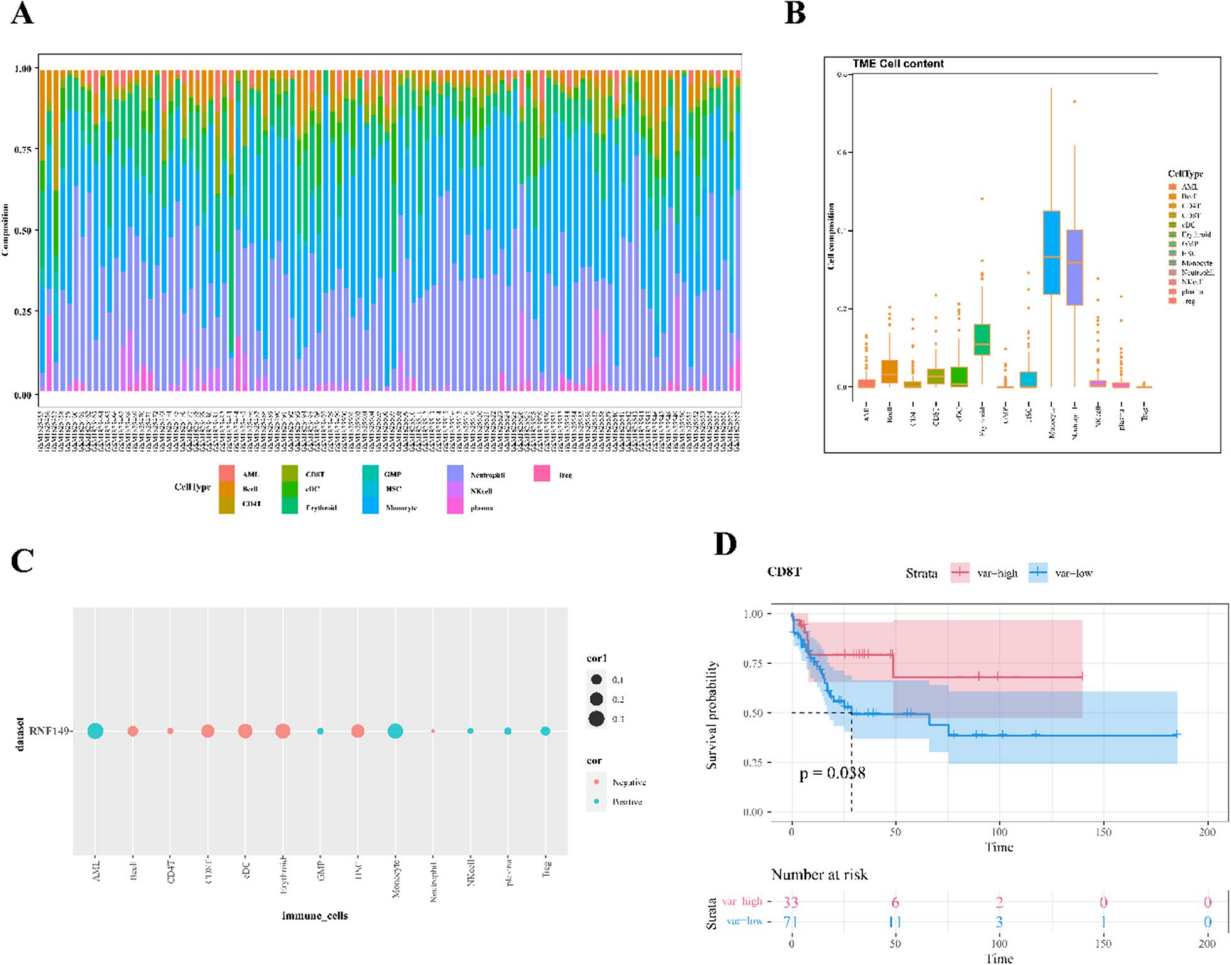
Role of RNF149 in modifying the tumor microenvironment (TME) of acute myeloid leukemia (AML). **A** Immune infiltration across samples; **B** Proportional cellular composition for all samples; **C** Association of RNF149 with cells within the bone marrow microenvironment; **D** Survival prognosis related to RNF149 expression in CD8^+^ T cells

To investigate the association between RNF149 and the tumor microenvironment (TME) of AML, we employed cibersortX, utilizing annotated scRNA data to examine the cellular components within the AML bone marrow microenvironment. Our analysis of the immune cell profiles across all AML patients revealed a distinct tumor heterogeneity (Fig. 7A). Notably, Monocytes and Neutrophils emerged as the predominant cellular components in the TME (Fig. 7B). We also established a correlation between RNF149 and the majority of cells infiltrating the AML-TME (Fig. 7C). Elevated RNF149 expression in CD8^+^T cells was associated with a poorer prognosis (Fig. 7D). Such findings imply that RNF149 may modulate the TME by inhibiting CD8^+^T cell functionality, consequently affecting AML prognosis negatively. Thus, understanding the immunosuppressive strategies employed by AML cells against CD8^+^ T cells and evaluating the interplay between tumor antigens within AML cells and TCR recognition is pivotal to the research on AML immune evasion and identification.

### RNF149 decreases the chemotherapeutic drug sensitivity in AML

**Fig. 8:**
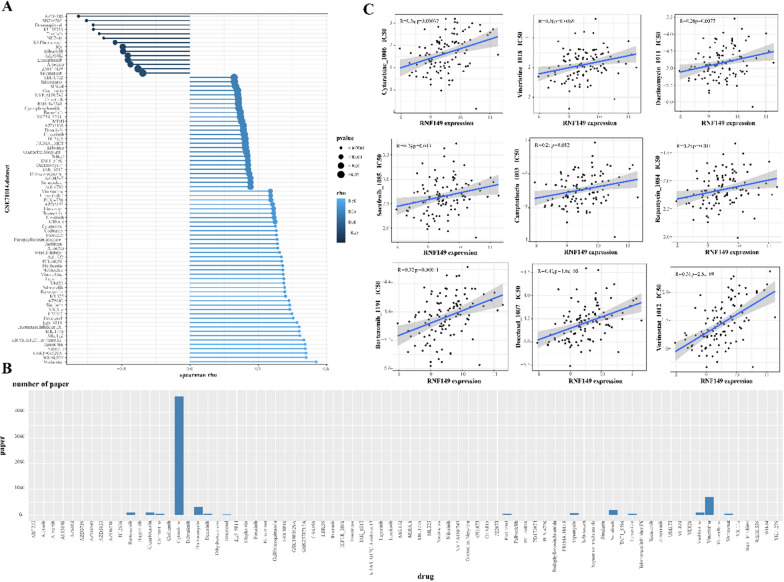
RNF149 and its association with multidrug resistance. **A** Spearman’s analysis of the correlation between risk scores and drug sensitivity. Each row corresponds to a drug. The length of each row signifies the degree of correlation, suggesting an association between the risk score and either drug resistance or sensitivity; **B** A count of published articles concerning 82 chemotherapy drugs used for acute myeloid leukemia (AML), sourced from PubMed; **C** IC50 values for nine chemotherapy drugs in AML patients expressing high levels of RNF149

To assess the drug sensitivity in AML patients exhibiting elevated RNF149 expression, we analyzed gene expression in AML tissues using the GSE71014 dataset. All scores for each sample, determined by the OncoPredict algorithm, are presented in Additional file 2: Table S2. Utilizing the OncoPredict package, we ascertained the IC50 values for prevalent drugs. Spearman correlation analysis revealed a relationship between the drug sensitivities of 82 antitumor agents and risk scores. Of these, 14 drugs demonstrated increased sensitivity in the high-risk cohort, whereas the remainder exhibited resistance (Fig. 8A). We subsequently employed a search function, based on MeSH terms, to quantify the literature on the aforementioned 82 chemotherapeutic agents in the context of AML, with cytarabine emerging as the most cited (Fig. 8B). Scatter plots were utilized to represent the IC50 values of 9 widely-prescribed chemotherapy drugs, suggesting that a higher IC50 corresponds to diminished sensitivity in high RNF149 expression samples and, consequently, less effective treatment outcomes (Fig. 8C).

### RNF149 potentially facilitates immune evasion in acute myeloid leukemia cells by suppressing CD8^+^T cell activity

**Fig. 9:**
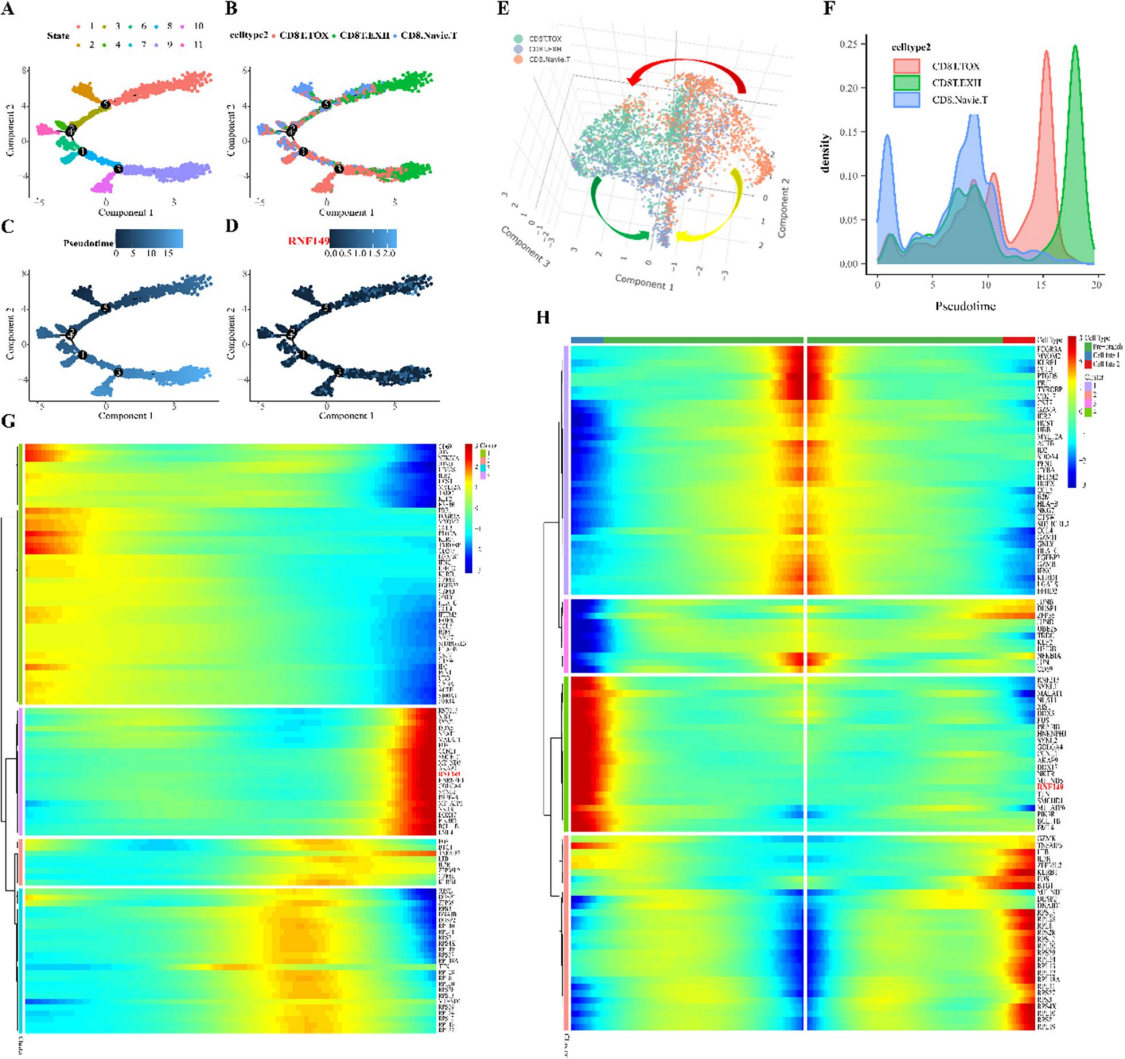
Role of RNF149 in Modulating CD8^+^ T Cell Evolution.**A**–**D**. Trajectories derived from Monocle 2 depict dynamic shifts in CD8^+^ T cell subclusters (**A**), subpopulations (**B**), temporal progression (**C**), and RNF149 expression (**D**). **E** Monocle 3 trajectory highlights CD8^+^ T cell subclusters. **F** Density plot delineating the temporal differentiation of three CD8^+^ T cell subpopulations. **G** Hierarchical clustering heatmap reveals marker genes linked with developmental phases and cell subpopulations. **H** Differential hierarchical clustering heatmap indicates marker genes pertinent to developmental stages and cellular subsets within differentiation lineage "branch-3."

Immune cells, particularly the activated CD8^+^T cells, play a pivotal role in the anti-tumor immune response. In the tumor microenvironment (TME) of patients with acute myeloid leukemia, multiple immune-suppressive mechanisms impede the efficacy of CD8^+^T cells. This impediment allows leukemia cells to bypass immune surveillance, thus contributing to disease progression and drug resistance. Upon isolating CD8^+^T cells and applying dimension reduction, clustering, and batch effect removal, we assessed the influence of different resolutions on cell clustering, as depicted in Additional file 1: Fig. S6A. We selected an optimal resolution and classified CD8^+^T cells into three primary categories: Naive CD8 T cells (CD8^+^.Naive.T), cytotoxic CD8^+^T cells (CD8^+^T_Tox_), and exhausted CD8^+^T cells (CD8^+^T_Exh_), as shown in Additional file 1: Fig. S6B. Predominantly, these cells are found in patients non-responsive to chemotherapy, with a minority present in those who responded, as highlighted in Additional file 1: Fig. S6C. In alignment with the bulk data, single-cell analysis revealed a heightened expression of RNF149 in CD8^+^T_Exh_ cells (Additional file 1: Fig. S6D). Markers such as SELL and IL7R are predominantly expressed in CD8^+^.Naive.T, GZMK and NKG7 in CD8^+^T_Tox_, and LAG3, HAVCR2, and RNF149 in CD8^+^T_Exh_, as shown in Additional file 1: Fig. S6E. Distinct expression profiles and biological functions are exhibited by CD8^+^T cells in varying states, as illustrated in Additional file 1: Fig. S6F. We employed the Monocle 2 and Monocle 3 algorithms to analyze the trajectory of CD8^+^T cells, aiming to decipher their spatiotemporal evolution in acute myeloid leukemia lesions. With Monocle 2, we organized cells along trajectories, presenting varying cell clusters, types, and differentiation durations in a two-dimensional representation, It’s evident that RNF149 is predominantly expressed in CD8^+^T_Exh_ cells (Fig. 9A–D). Monocle 3 results distinctly delineate the differentiation pathways from CD8^+^.Naive.T to CD8^+^T_Tox_ and CD8^+^T_Exh_, as well as the evolution process from CD8^+^T_Tox_ to CD8^+^T_Exh_ (Fig. 9E). Investigating the gene dynamics during the CD8^+^T cell evolution process is imperative. Throughout this process, RNF149 initially diminishes before surging. Notably, the escalation in RNF149 coincides with an extensive accumulation of cells in the direction of CD8^+^TExh (Fig. 9F, G). Single-cell trajectory branches emerge due to cells adopting divergent gene expression patterns. At branching point 3, CD8^+^T cells embark on one of two trajectories: either they differentiate towards CD8^+^T_Tox_ or predominantly evolve into CD8^+^TExh. Intriguingly, a marked increase in RNF149 is observed when cells differentiate towards CD8^+^T_Exh_ (Fig. 9H).

### Evaluation of RNF149 expression

**Fig. 10:**
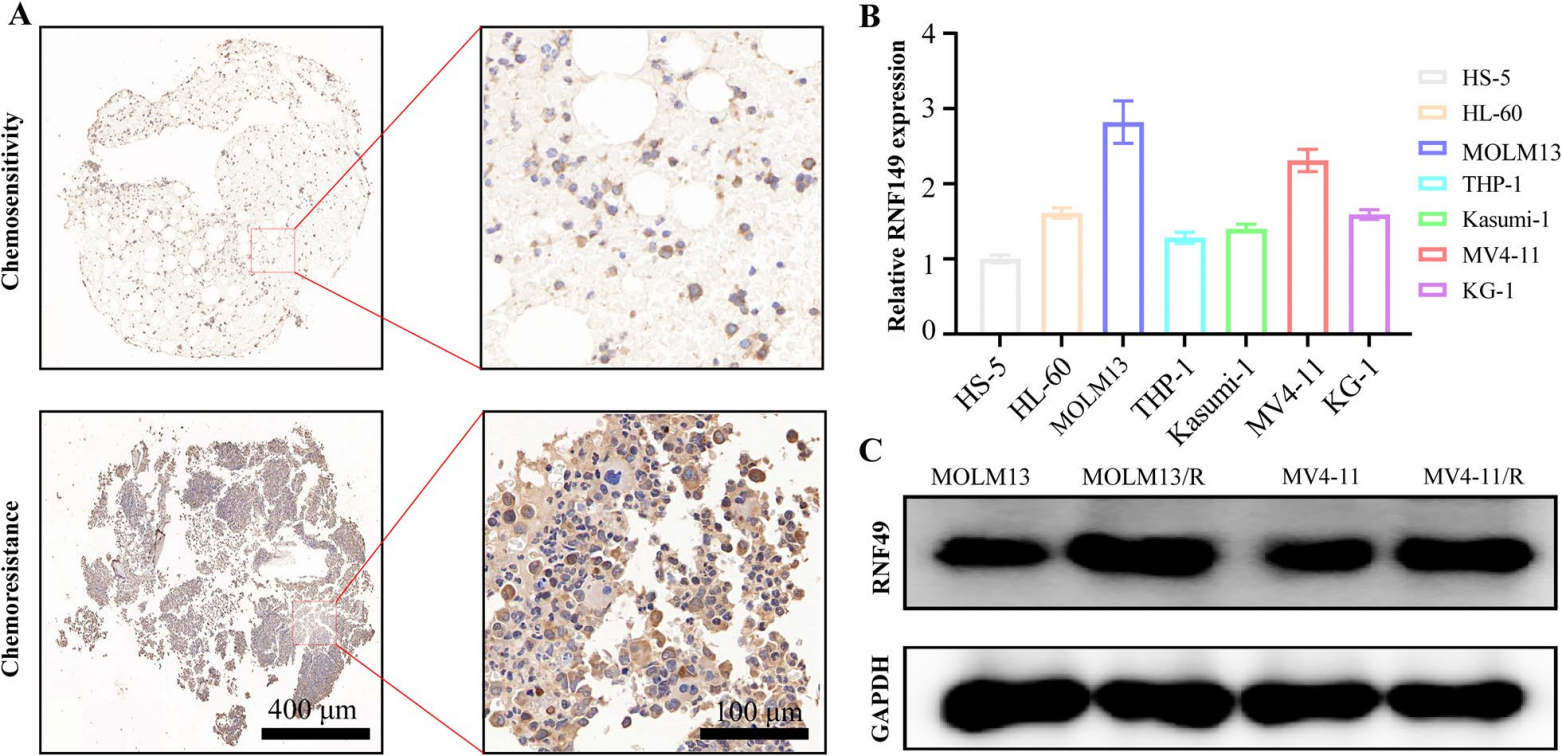
RNF149 Expression in AML Patient Tissues and Cell Lines. **A** Immunohistochemical analysis of RNF149 in bone marrow tissues from AML patients in chemotherapy remission and non-remission; **B** qRT-PCR assessment of RNF149 levels in AML cell lines compared to the normal control HS-5 cells; **C** Western blot quantification of RNF149 in MOLM13, MV4-11, MOLM13/R, and MV4-11/R cell lines

Immunohistochemistry revealed diminished RNF149 expression in the bone marrow tissues of post-chemotherapy AML patients in remission compared to those without remission (Fig. 10A). An examination of RNF149 mRNA levels in MM cell lines HL-60, MOLM13, THP-1, Kasumi-1, MV4-11, KG-1, and the control cell HS-5 demonstrated that the expression in AML cell lines was notably elevated compared to HS-5 cells, particularly in MOLM13 and MV4-11 (Fig. 10B). This research also established two AML cell line models resistant to cytarabine through a gradient increment technique (Additional file 1: Fig. S7A). An analysis of the IC50 values for MOLM13 and MV4-11 cell lines indicated respective values of 4.79 µmol/L and 1.56 µmol/L for cytarabine. Employing a stepwise dosage increase, resistant cell lines, termed MOLM13/R and MV4-11/R, were developed, both exceeding an IC50 of 1000 µmol/L (Additional file 1: Fig. S7B). Western blotting indicated a marked reduction in RNF149 protein levels in MOLM13 and MV4-11 cells compared to their drug-resistant counterparts, MOLM13/R and MV4-11/R (Fig. 10C and Additional file 1: Fig. S8A).

### Silencing of RNF149 suppresses Acute myeloid leukemia cell proliferation

**Fig. 11:**
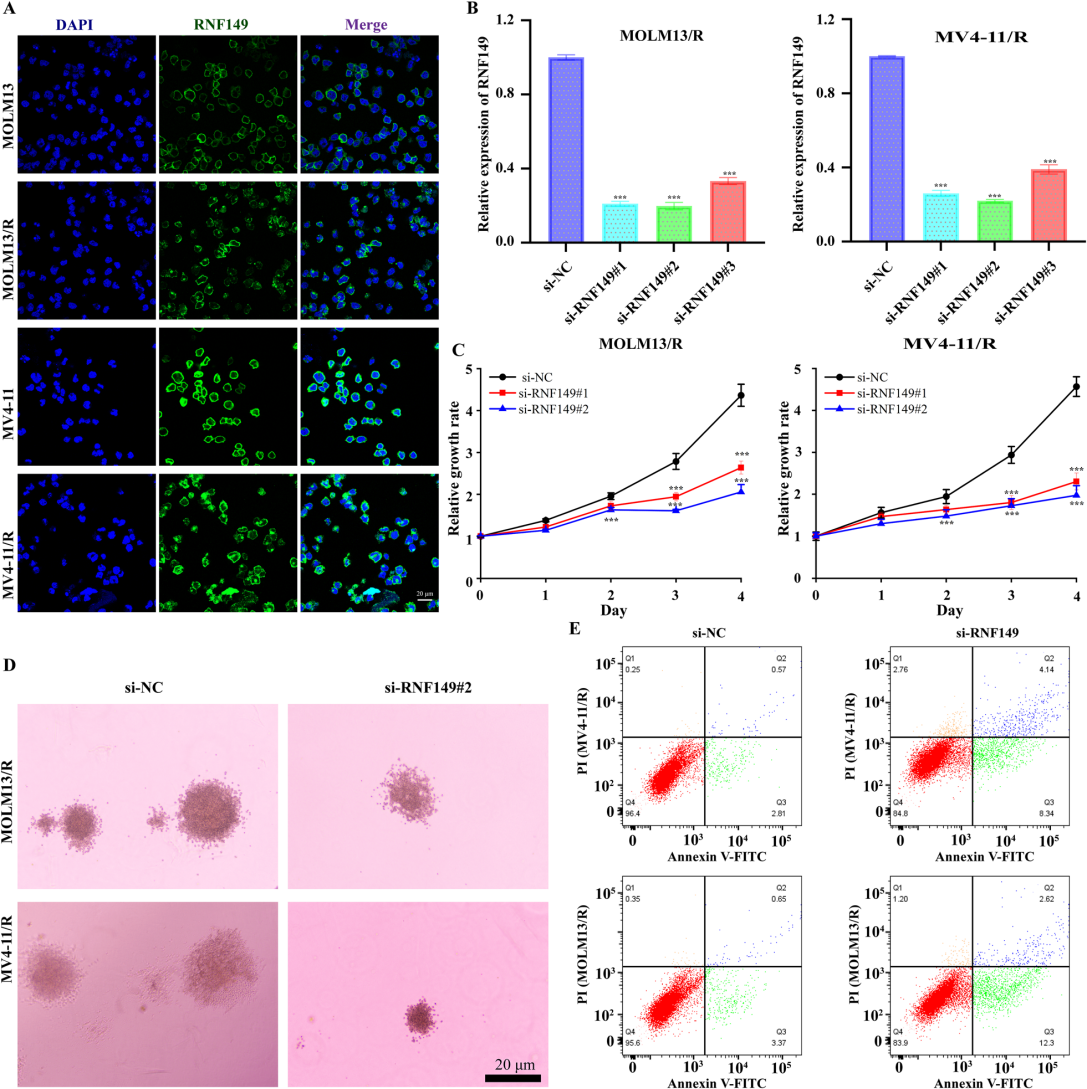
Intracellular localization of RNF149 and its function in AML drug-resistant cells. **A** Immunofluorescence demonstrates the presence of RNF149 in both AML and drug-resistant cells, with nuclei stained using DAPI (scale bar: 20 μm); **B** Efficiency of RNF149 knockdown is verified in MOLM13/R and MV4-11/R cells. Subsequent assays include CCK-8 **C**, colony formation **D**, and flow cytometry for apoptosis detection **E** to ascertain the role of RNF149 in AML drug-resistant cells. Data are represented as mean ± SD (n = 3), with intergroup comparisons indicated as: * p < 0.05, ** p < 0.01, and *** p < 0.001

To determine RNF149’s localization within AML cells, we conducted further tests. Immunofluorescence data revealed that RNF149 is present on both the cell membrane and within the cytoplasm of MOLM13 and MV4-11 cells. In contrast, in MOLM13/R and MV4-11/R cells, RNF149 protein predominantly resides in the cytoplasm, with varied migration into the nucleus (Fig. 11A). We constructed three specific siRNAs (si-RNF149#1, si-RNF149#2, and si-RNF149#3) to investigate RNF149’s proliferative function in AML cells. Results from the CCK-8 assay and cell colony formation tests indicated that the silencing of RNF149 suppressed its expression in MOLM13/R and MV4-11/R cells, thereby inhibiting cell proliferation (Fig. 11B–D and Additional file 1: Fig. S8B). Following RNF149 silencing, flow cytometry analysis indicated a notable enhancement in cell apoptosis, especially with si-RNF149#2 (Fig. 11E and Additional file 1:  Fig. S8C). In summary, our findings suggest RNF149 plays a vital role in promoting AML cell growth and proliferation.

### Biological Implications of RNF149: in vivo analysis

**Fig. 12:**
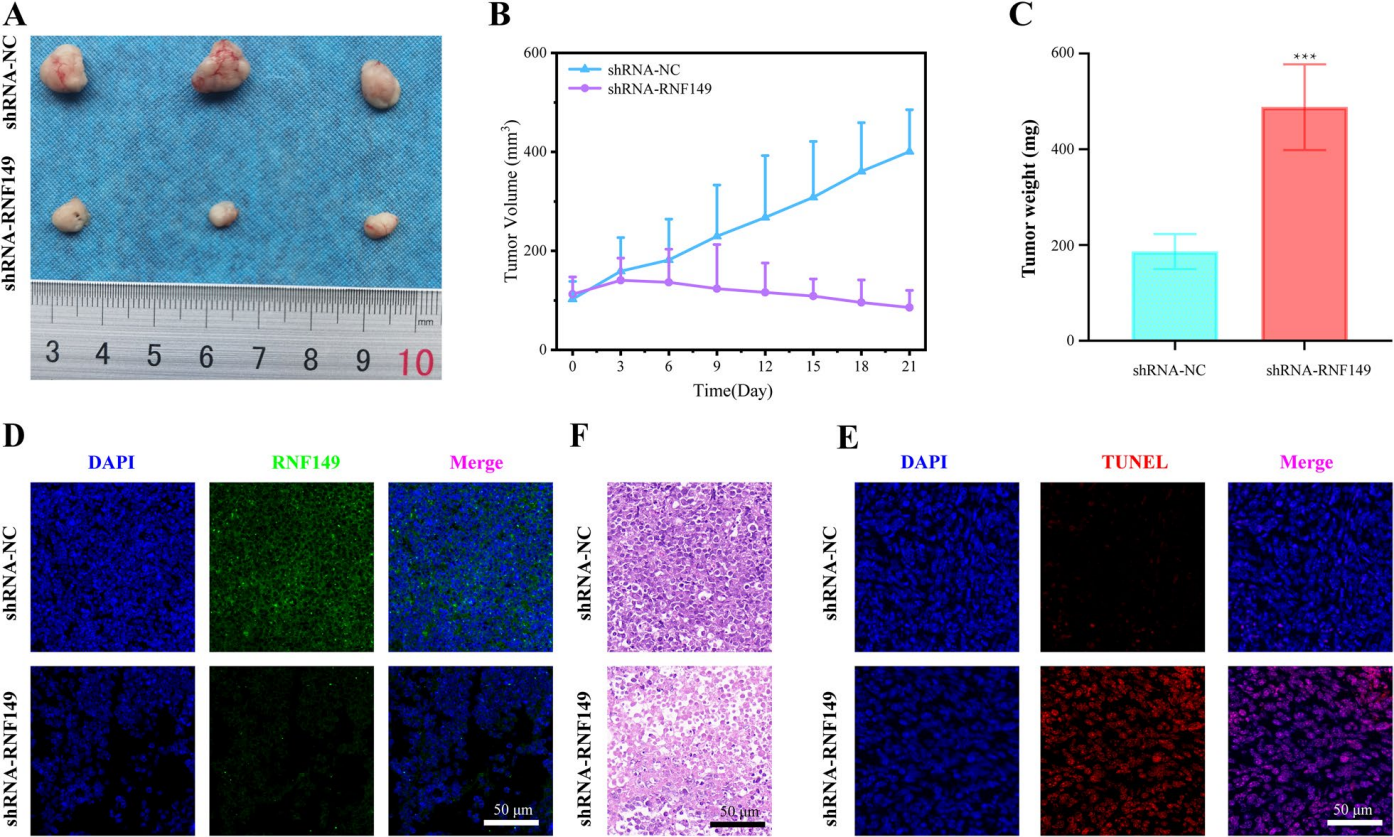
In vivo validation of RNF149's tumorigenic role. **A** Dissected mouse tumor tissue; **B**–**C** Tumor volume and weight across groups; **D** RNF149 expression in tumor tissues analyzed by immunofluorescence; **E**–**F** TUNEL assay and H&E staining of tumor tissues from various groups

With the progression of days post-tumor inoculation, the shRNA-NC group mice exhibited a consistent growth in tumor volume. Conversely, the RNF149-shRNA group demonstrated a significant decline in tumor volume relative to the shRNA-NC group (Fig. 12A, B). Subsequent analyses revealed a notable reduction in tumor mass within the shRNA-RNF149 cohort compared to the shRNA-NC group (Fig. 12C). Immunofluorescence studies indicated diminished green fluorescence intensity in the tumor tissues of the shRNA-RNF149 mice, reflecting a reduced expression of the RNF149 protein when contrasted with the NC-shRNA group (Fig. 12D). TUNEL staining unveiled an elevated number of apoptosis-indicating TUNEL positive cells (red fluorescence) in the shRNA-RNF149 group, a marked increase from the shRNA-NC group. Moreover, HE-stained tumor sections revealed decelerated tumor progression in the shRNA-RNF149 cohort relative to the shRNA-NC group (Fig. 12E). Collectively, these findings suggest that RNF149 inhibition may enhance the growth and apoptosis of subcutaneous AML tumors.

## Discussion

AML progresses swiftly, presenting a bleak prognosis, its pathogenesis remains elusive [23]. Contemporary research suggests that the genesis and evolution of AML might intimately correlate with the immune microenvironment within its bone marrow [24]. This association may extend to AML’s therapeutic responsiveness. Utilizing single-cell sequencing, one can probe the prevalence and functions of disparate immune cell subsets in both the AML-afflicted bone marrow environments [25]. Such exploration facilitates a comprehensive understanding of the constitution and operational status of AML bone marrow cells, enriching insights into the AML bone marrow immune microenvironment [26]. This ultimately furnishes a theoretical scaffold for AML management. In this study, an examination of AML single-cell datasets from the GEO database was undertaken, utilizing the R language’s Seurat package to categorize 121,383 bone marrow cells into 13 predominant cell categories. By juxtaposing AML patients post-chemotherapy remission with non-remissive counterparts, discernible shifts in the bone marrow’s microenvironmental components became evident, highlighting the intrinsic heterogeneity of AML cells.

Chemotherapy-resistant or relapsed AML remains a therapeutic challenge [27]. A central aim in both foundational and applied research is the development of strategies to extend the disease-free survival of patients [28]. Current research underscores the role of T-cell immune homeostasis disruption within the AML bone marrow microenvironment in AML’s etiology and progression [29]. This imbalance not only influences patient prognosis but also offers insights for novel immunotherapeutic approaches [30]. T-cell exhaustion within the bone marrow significantly contributes to leukemia’s ability to evade immune responses and subsequently affects patient outcomes [31]. Such exhaustion is an immunosuppressive state arising from persistent tumor antigen stimulation [32]. Co-stimulatory signals from myeloid leukemia cells enhance Th1 cell exhaustion, elevating immune checkpoints such as PD1, TIM-3, LAG-3, and CTLA-4. This upregulation diminishes the release of IL-2, TNF-α, and IFN-γ, culminating in T-cell exhaustion. Exhausted T cells (Tex) are pivotal in tumor evolution [33]. They attenuate host immunity, facilitating tumor immune evasion and aggression, thereby promoting tumorigenesis. CD4^+^T cells and CD8^+^T cells serve as linchpins in human immunoregulation [34]. The former discerns exogenous antigenic peptides via MHC class II molecules, whereas the latter identifies endogenous peptides through MHC class I molecules [35]. This study’s single-cell sequencing results reveal that the AML group’s T/CD8^+^ T cells display compromised protein synthesis and immune functionality, including reduced cytoplasmic translation and ribosomal biosynthesis. This suggests that these T cells in the AML cohort exhibit diminished cytotoxicity against leukemia cells, leading to immune dysfunction and loss of monitoring capabilities, which in turn accelerates leukemia progression.

Hematologic malignancies commonly arise from alterations in various genes and protein functionalities [36]. Protein ubiquitination, a pivotal post-translational modification, is central to regulating diverse physiological processes, with the E3 ubiquitin ligase being paramount [37]. Through integrating scRNA-seq and Bulk-seq databases, we discerned a notably overexpressed E3 ligase, RNF149, in acute myeloid leukemia (AML), which correlates with survival outcomes. Employing bioinformatic tools, including GO, KEGG, GSVA, and GSEA, we elucidated RNF149’s prospective roles in facilitating cancerous growth, impeding immune response, modulating cellular signaling, and fostering drug resistance in AML. Significantly, our assessment of chemotherapeutic drug susceptibility indicates that RNF149 not only elevates the IC50 values of various drugs in AML but also demonstrates considerable affinity to primary chemotherapy agents. Our in vitro and in vivo assessments revealed that RNF149 impedes apoptosis in AML cells, augments their proliferation, and mirrors this function in drug-resistant AML subsets and murine tumor models. This underscores RNF149’s pivotal role in AML’s resistance to cytarabine treatment. Importantly, CD8^+^T cells, being the immune response’s primary effector cells, can selectively annihilate target cells. Within the milieu of somatic cell metamorphosis, these CD8T cells pioneer the recognition and elimination of neoplastic cells. Yet, within AML’s intricate immune microenvironment, these cells frequently exhibit signs of functional fatigue, characterized by diminished efficacy and replicative potential, implicating their central role in AML recurrence and drug resistance. Our pseudo-temporal analysis charted the path of CD8^+^T cells, revealing RNF149’s predominant expression in CD8^+^T_Exh_ cells, suggesting its role in steering the transition from CD8^+^.Navie.T to CD8^+^T_Exh_ in AML.

## Conclusions

In conclusion, RNF149 is implicated in fostering drug resistance in acute myeloid leukemia by both enhancing the proliferation of resistant cells and reducing their sensitivity to cytarabine. Notably, RNF149 has been observed to trigger functional exhaustion in CD8^+^T cells, thereby driving their transition to CD8^+^T_Exh_, which subsequently disrupts the immune equilibrium within the AML microenvironment. The emergence of drug resistance in leukemia cells stems from a synergy of diverse pathways and molecular mechanisms. Further investigation into RNF149’s role in AML drug resistance is imperative to identify novel therapeutic targets and to underpin the development of efficacious and safe treatment modalities.

### Supplementary Information

**Additional file 1: Table S1.** Primers used for the qRT-PCR analysis. **Figure S1.** Quality control analysis for the acute myeloid leukemia single database. **A** Identification of genes with high variability across cells; x-axis represents average expression and y-axis denotes normalized variance. **B** PCA representation colored according to 93 individual samples. **C** PCA depiction of cell cycle distribution across the 93 samples. **D** Scatter plot correlating overall gene expression in cells with the proportion of mitochondrial genes. **E** Scatter plot comparing overall gene expression in cells with gene counts percentage. **F**–**G** Violin plots illustrating gene counts and total gene expression levels, respectively, for each sample. **H**–**I** Violin plots presenting G2M and S phase score levels for each sample, respectively. **Figure S2.** Dot plot illustrating acute myeloid leukemia cells influencing other cell subgroups. **Figure S3.** Analysis of communication probability modulated by ligand-receptor pairs between acute myeloid leukemia cells and other cellular groups. **Figure S4.** Validation of the prognostic potential of AML-E3s using the GSE106291 dataset. **Figure S5.** Confirmation of the prognostic potential of AML-E3s with the GSE71014 dataset. **Figure S6.** CD8+T cell dimensionality reduction, clustering, annotation, and subgroup analysis. **A** Dendrogram illustrating clustering across varied resolutions; **B** UMAP representation of chosen resolution and cell categories; **C** UMAP visualization of distinct clinical type distributions among CD8+T cell subgroups; **D** UMAP portrayal of RNF149 expression across CD8+T cell subgroups; **E** Marker gene dot plot; **F** DEGs expression heatmap in three CD8+T cell sub-clusters. **Figure S7.** **A** Diagram of AML drug-resistant cell line development; **B** Enhanced cytarabine resistance observed in MOLM13/R and MV4-11/R cell lines. **Figure S8.** **A** Quantification of RNF149 protein levels via ImageJ software; **B** Post-transfection cell colony counts for MOLM13/R and MV4-11/R cells using si-NC and si-RNF149, 14 days post-procedure, utilizing ImageJ software; **C** Analysis of cell apoptosis rates. ***p<0.001 compared to si-NC

**Additional file 2.** Analysis of predicted drug sensitivity to various drugs in each patient.

## Data Availability

All data generated or analyzed during this study are included in this published article.
