# Supplementary material for: Spatiotemporal evolution of AML immune microenvironment remodeling and RNF149-driven drug resistance through single-cell multidimensional analysis
Source: J Transl Med. 2023 Oct 27;21:760. doi: 10.1186/s12967-023-04579-5 (PMC10612211; doi:10.1186/s12967-023-04579-5)
Supplement: Supplementary file 1 — Additional file 1: Table S1. Primers used for the qRT-PCR analysis. Figure S1. Quality control analysis for the acute myeloid leukemia single database. A Identification of genes with high variability across cells; x-axis represents average expression and y-axis denotes normalized variance. B PCA representation colored according to 93 individual samples. C PCA depiction of cell cycle distribution across the 93 samples. D Scatter plot correlating overall gene expression in cells with the proportion of mitochondrial genes. E Scatter plot comparing overall gene expression in cells with gene counts percentage. F–G Violin plots illustrating gene counts and total gene expression levels, respectively, for each sample. H–I Violin plots presenting G2M and S phase score levels for each sample, respectively. Figure S2. Dot plot illustrating acute myeloid leukemia cells influencing other cell subgroups. Figure S3. Analysis of communication probability modulated by ligand-receptor pairs between acute myeloid leukemia cells and other cellular groups. Figure S4. Validation of the prognostic potential of AML-E3s using the GSE106291 dataset. Figure S5. Confirmation of the prognostic potential of AML-E3s with the GSE71014 dataset. Figure S6. CD8+T cell dimensionality reduction, clustering, annotation, and subgroup analysis. A Dendrogram illustrating clustering across varied resolutions; B UMAP representation of chosen resolution and cell categories; C UMAP visualization of distinct clinical type distributions among CD8+T cell subgroups; D UMAP portrayal of RNF149 expression across CD8+T cell subgroups; E Marker gene dot plot; F DEGs expression heatmap in three CD8+T cell sub-clusters. Figure S7. A Diagram of AML drug-resistant cell line development; B Enhanced cytarabine resistance observed in MOLM13/R and MV4-11/R cell lines. Figure S8. A Quantification of RNF149 protein levels via ImageJ software; B Post-transfection cell colony counts for MOLM13/R and MV4-11/R cells using si-NC and [file 12967_2023_4579_MOESM1_ESM.doc]

**Additional materials**

| **Table S1.** Primers used for the qRT-PCR analysis. | |
| --- | --- |
| Primer name | Sequence (5'→3') |
| RNF149-forward | AACATCACCTTGCCCATGTCT |
| RNF149-reverse | CCCCTATGGTCATCGTTACTGG |
| GAPDH-forward | GTCTCCTCTGACTTCAACAGCG |
| GAPDH-reverse  si-RNF149#1  si-RNF149#2  si-RNF149#3  Homo-RNF149-shRNA | ACCACCCTGTTGCTGTAGCCAA  GCUUCUACUUCAUACUGUAAA  GAACAUGUCCAAUGUGUAAAC  GGAGAUGCCUGCUCCAGAAUC  CATCAAAGCCCTAGGATATTG |


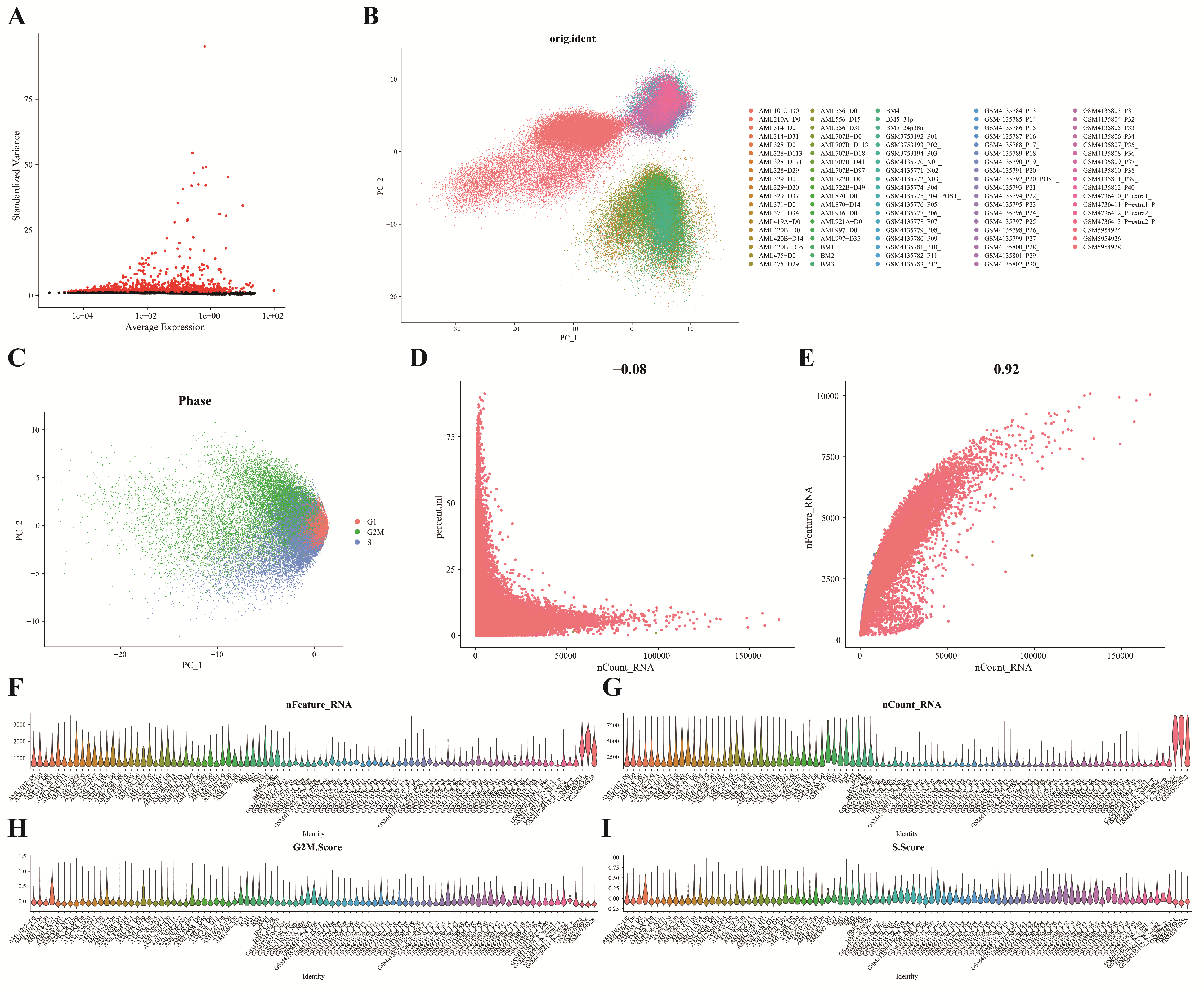


**Additional Figure 1.** Quality control analysis for the acute myeloid leukemia single database. (A) Identification of genes with high variability across cells; x-axis represents average expression and y-axis denotes normalized variance. (B) PCA representation colored according to 93 individual samples. (C) PCA depiction of cell cycle distribution across the 93 samples. (D) Scatter plot correlating overall gene expression in cells with the proportion of mitochondrial genes. (E) Scatter plot comparing overall gene expression in cells with gene counts percentage. (F-G) Violin plots illustrating gene counts and total gene expression levels, respectively, for each sample. (H-I) Violin plots presenting G2M and S phase score levels for each sample, respectively.


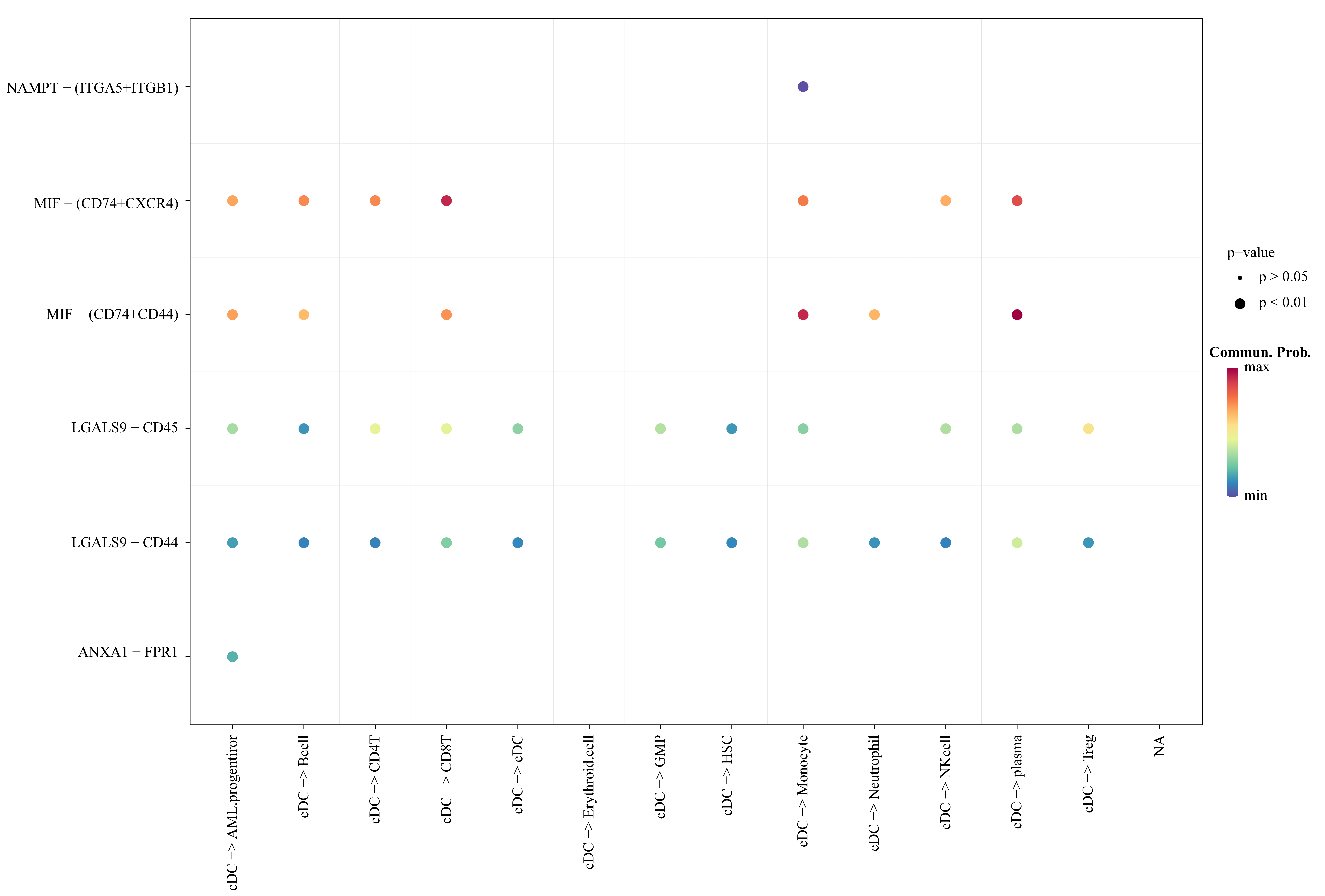


**Additional Figure 2.** Dot plot illustrating acute myeloid leukemia cells influencing other cell subgroups.


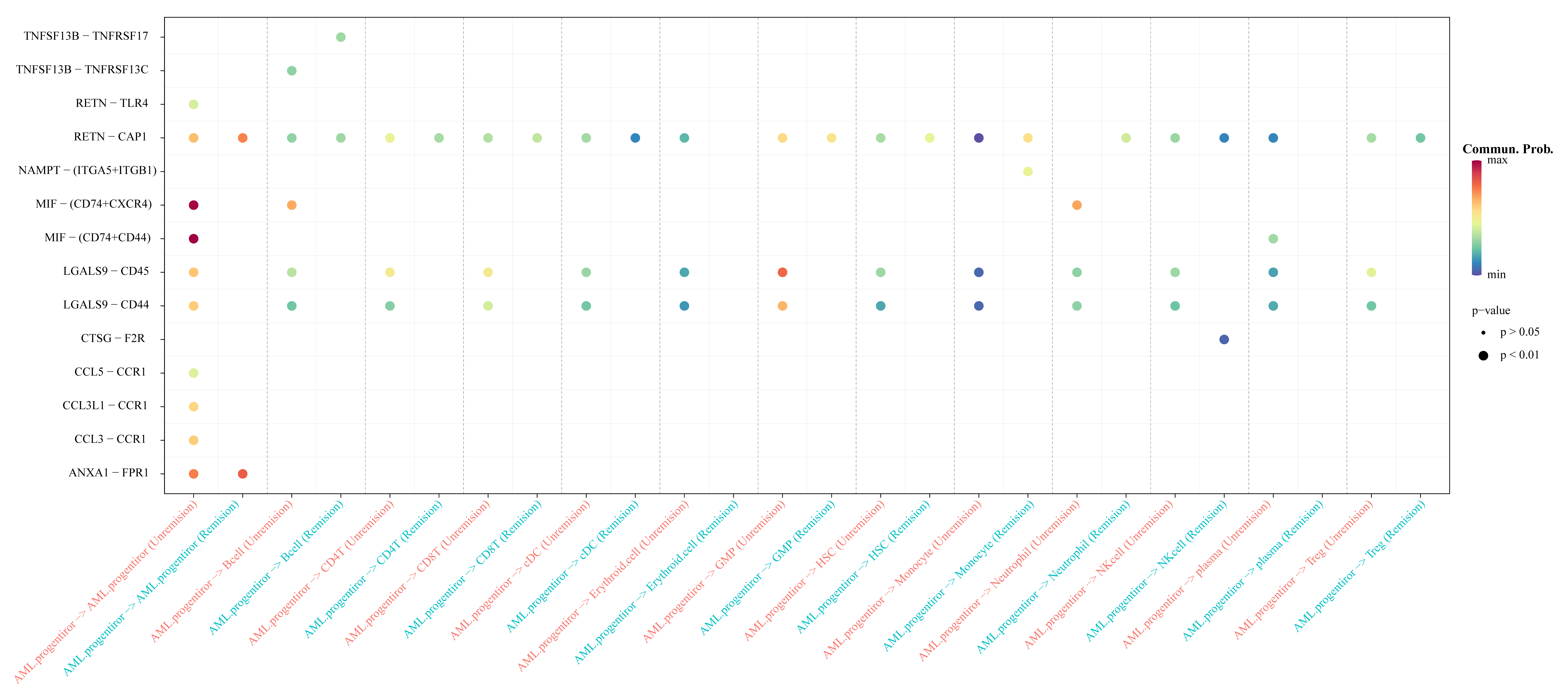


**Additional Figure 3.** Analysis of communication probability modulated by ligand-receptor pairs between acute myeloid leukemia cells and other cellular groups.





**Additional Figure 4.** Validation of the prognostic potential of AML-E3s using the GSE106291 dataset.





**Additional Figure 5.** Confirmation of the prognostic potential of AML-E3s with the GSE71014 dataset.


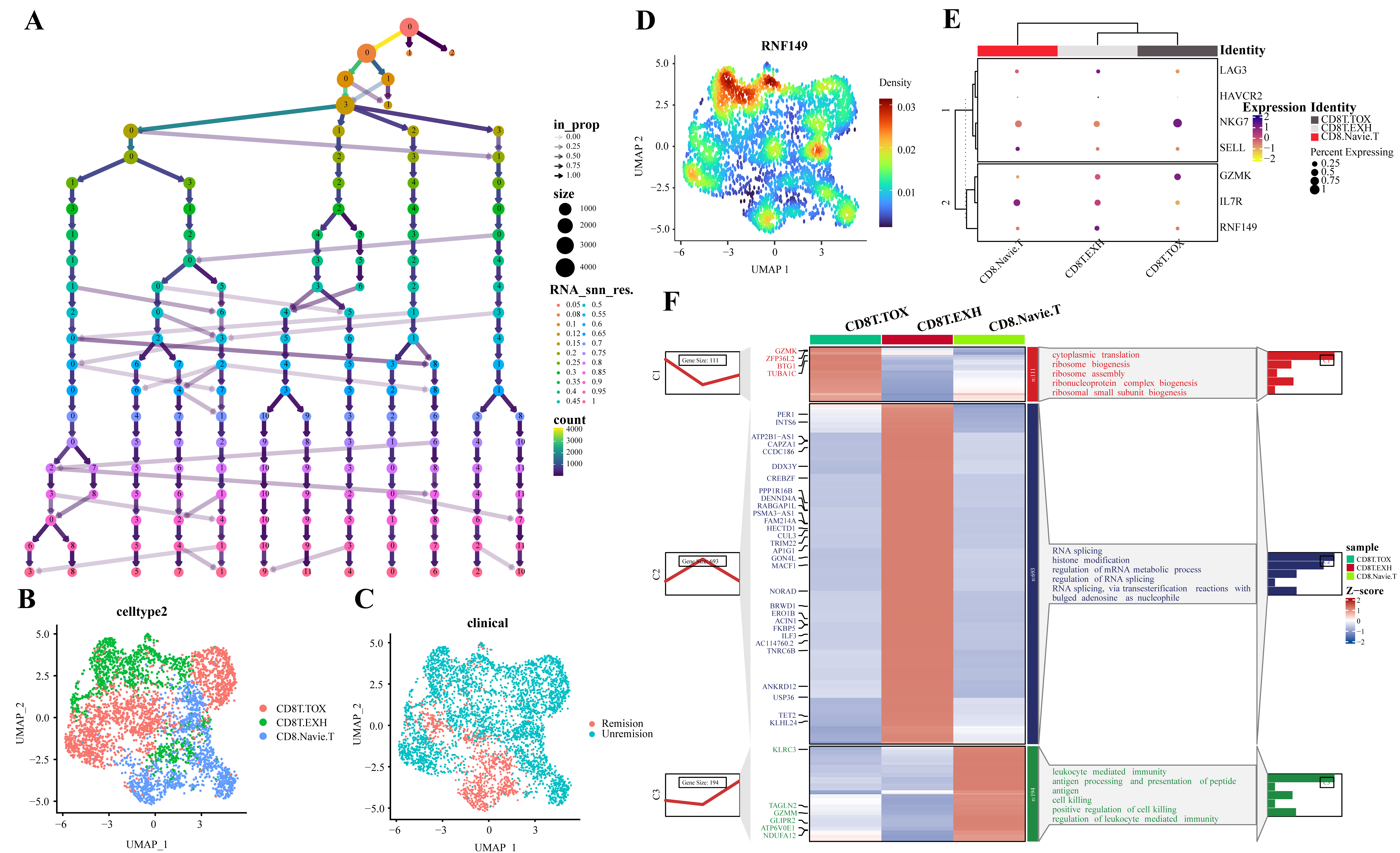


**Additional Figure 6.** CD8^+^T cell dimensionality reduction, clustering, annotation, and subgroup analysis. A. Dendrogram illustrating clustering across varied resolutions; B. UMAP representation of chosen resolution and cell categories; C. UMAP visualization of distinct clinical type distributions among CD8^+^T cell subgroups; D. UMAP portrayal of RNF149 expression across CD8^+^T cell subgroups; E. Marker gene dot plot; F. DEGs expression heatmap in three CD8^+^T cell sub-clusters.


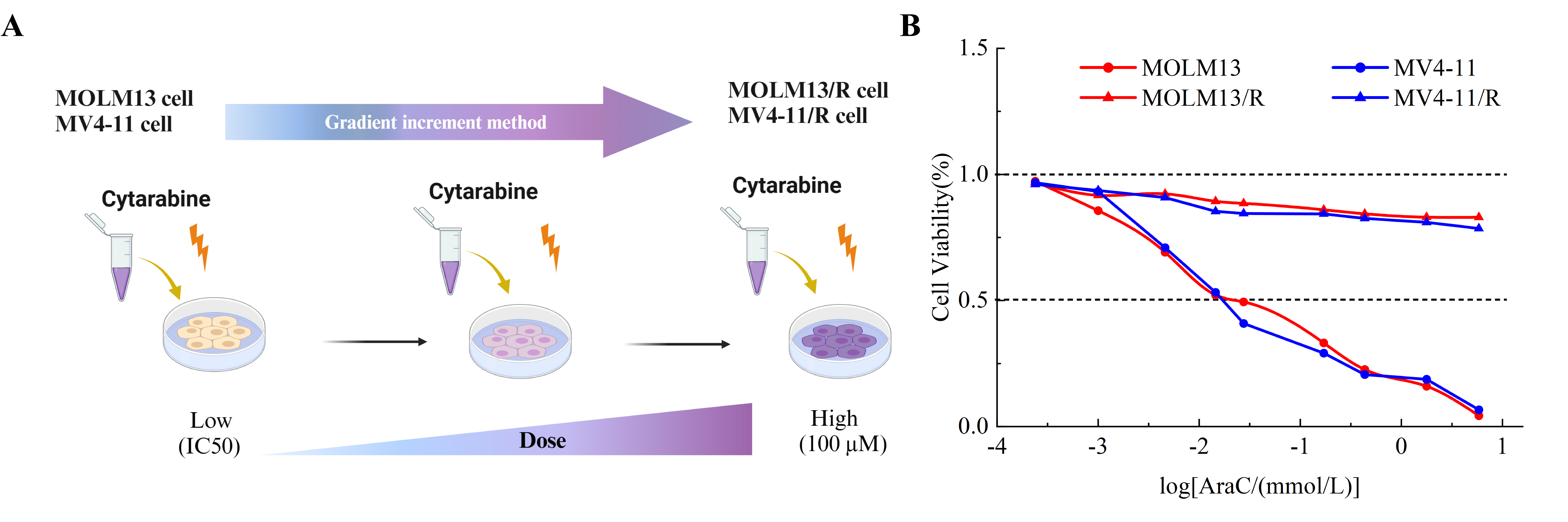


**Additional Figure 7. (A) Diagram of AML drug-resistant cell line development; (B) Enhanced cytarabine resistance observed in MOLM13/R and MV4-11/R cell lines.**

**
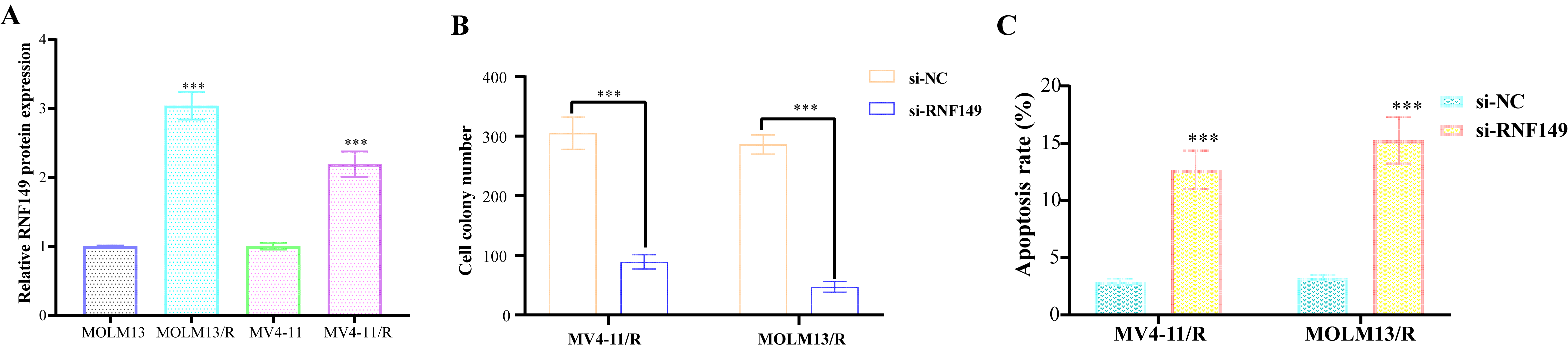
Supplementary Figure 8.** (A) Quantification of RNF149 protein levels via ImageJ software; (B) Post-transfection cell colony counts for MOLM13/R and MV4-11/R cells using si-NC and si-RNF149, 14 days post-procedure, utilizing ImageJ software; (C) Analysis of cell apoptosis rates. ***p<0.001 compared to si-NC.
